# Divergent brain gene expression patterns associate with distinct cell-specific tau neuropathology traits in progressive supranuclear palsy

**DOI:** 10.1007/s00401-018-1900-5

**Published:** 2018-08-22

**Authors:** Mariet Allen, Xue Wang, Daniel J. Serie, Samantha L. Strickland, Jeremy D. Burgess, Shunsuke Koga, Curtis S. Younkin, Thuy T. Nguyen, Kimberly G. Malphrus, Sarah J. Lincoln, Melissa Alamprese, Kuixi Zhu, Rui Chang, Minerva M. Carrasquillo, Naomi Kouri, Melissa E. Murray, Joseph S. Reddy, Cory Funk, Nathan D. Price, Todd E. Golde, Steven G. Younkin, Yan W. Asmann, Julia E. Crook, Dennis W. Dickson, Nilüfer Ertekin-Taner

**Affiliations:** 1 Department of Neuroscience, Mayo Clinic, Jacksonville, FL 32224 USA; 2 Department of Health Sciences Research, Mayo Clinic, Jacksonville, FL 32224 USA; 3 Division of Information Technology, Mayo Clinic, Jacksonville, FL 32224 USA; 4 Banner Behavior Health, Phoenix, AZ 85016 USA; 5 The Center for Innovation in Brain Sciences, University of Arizona, Tucson, AZ 85721 USA; 6 Department of Neurology, University of Arizona, Tucson, AZ 85721 USA; 7 Institute for Systems Biology, 401 Terry Avenue N, Seattle, WA 98109 USA; 8 Department of Neuroscience, Center for Translational Research in Neurodegenerative Disease, McKnight Brain Institute, University of Florida, Gainesville, FL 32610 USA; 9 Department of Neurology, Mayo Clinic, 4500 San Pablo Road, Birdsall 3, Jacksonville, FL 32224 USA

## Abstract

Progressive supranuclear palsy (PSP) is a neurodegenerative parkinsonian disorder characterized by tau pathology in neurons and glial cells. Transcriptional regulation has been implicated as a potential mechanism in conferring disease risk and neuropathology for some PSP genetic risk variants. However, the role of transcriptional changes as potential drivers of distinct cell-specific tau lesions has not been explored. In this study, we integrated brain gene expression measurements, quantitative neuropathology traits and genome-wide genotypes from 268 autopsy-confirmed PSP patients to identify transcriptional associations with unique cell-specific tau pathologies. We provide individual transcript and transcriptional network associations for quantitative oligodendroglial (coiled bodies = CB), neuronal (neurofibrillary tangles = NFT), astrocytic (tufted astrocytes = TA) tau pathology, and tau threads and genomic annotations of these findings. We identified divergent patterns of transcriptional associations for the distinct tau lesions, with the neuronal and astrocytic neuropathologies being the most different. We determined that NFT are positively associated with a brain co-expression network enriched for synaptic and PSP candidate risk genes, whereas TA are positively associated with a microglial gene-enriched immune network. In contrast, TA is negatively associated with synaptic and NFT with immune system transcripts. Our findings have implications for the diverse molecular mechanisms that underlie cell-specific vulnerability and disease risk in PSP.

**Electronic supplementary material:**

The online version of this article (10.1007/s00401-018-1900-5) contains supplementary material, which is available to authorized users.

## Introduction

Intracellular aggregation of hyperphosphorylated tau protein is a common neuropathological feature of many neurodegenerative diseases including Alzheimer’s disease (AD), progressive supranuclear palsy (PSP), corticobasal degeneration (CBD), and Pick’s disease (PiD) [39]. However, these tauopathies differ with respect to the aggregated isoform of tau protein, the distribution and localization of tau pathology, and affected central nervous system (CNS) cell types. PSP is a progressive parkinsonian disorder, estimated to affect approximately five people in every 100,000 [10], with a typical age of onset of greater than 60 years [14]. Four characteristic tau lesions observed in PSP brains are coiled bodies (CB), neurofibrillary tangles (NFT), tufted astrocytes (TA) and tau threads (TAUTh). These represent both neuronal and glial neuropathology where NFTs are found in neurons, CB in oligodendroglia, TA in astrocytes, and TAUTh represent filamentous tau threads present in white matter [17]. Although the predominant tau isoform in all PSP lesions has four microtubule-binding domains (4R), the four distinct tau lesions have morphological and/or biochemical differences [17, 54], indicative of possible cell-specific pathomechanisms. Common genetic variants at seven loci were reported to associate with risk in a PSP GWAS [26]. Some of these PSP risk variants are also associated with both brain expressions of proximal genes, and PSP neuropathology, implicating transcriptional regulation in disease etiology [2, 26, 58]. With the exception of these prior studies focused on expression of PSP candidate genes, little is currently known of other transcriptional changes in the human brain that may be implicated in PSP risk and the distinct tau neuropathological lesions observed in this disease.

We hypothesize that brain transcriptional changes underlie neuropathology in PSP. Therefore, transcriptome-wide association analysis of brain gene levels and PSP neuropathologic traits can identify molecular mechanisms that are both commonly and uniquely involved in the four distinct PSP tau lesions. To identify the genes and biological pathways that may underlie the distinct neuropathological features and thereby disease risk in PSP, we measured brain transcriptome levels in two independent cohorts collectively composed of 268 autopsy-confirmed PSP cases. Expression measurements were obtained from the temporal cortex, a region relatively preserved from PSP neuropathology [17] to reduce possible confounding of transcript levels from cell-type variability as a consequence of local neuropathology [48]. All patients also had continuous quantitative measures for the four distinct tau neuropathologies and a measure of overall tau burden generated from semi-quantitative tau pathology counts from 19 brain regions [2].

We performed transcriptome-wide association analyses of both individual genes and co-expression networks [34] with the four PSP neuropathology traits and identified both common and divergent patterns of association for the distinct tau lesions. Neuropathology-associated genes and networks were enriched for numerous biological pathways including synaptic and immune processes. These had opposite patterns of association with TA vs. NFT pathologies, implicating distinct pathomechanisms in astrocytes vs. neurons in PSP. The most significant neuronal co-expression network enriched for synaptic genes also had enrichment for PSP candidate risk genes. This suggests that genetic variants may influence PSP risk via their effects on regulatory networks. All significant co-expression networks had significant overrepresentation of genes located on certain chromosomes and also harbored strongly connected transcription factors, which suggests biological co-regulation of these transcripts.

## Methods

### Studies and subjects

All PSP subjects described in this study are from the Mayo Clinic Brain Bank, received a neuropathological diagnosis at autopsy [24], and belong to prior transcriptome studies which have been previously described. There are two independent cohorts with microarray expression data, designated as “Cohort A”, comprised of 173 PSP subjects [2] and “Cohort B”, comprised of 95 PSP subjects [2, 60], after quality control (QC) suppl. text (Online Resource 3). We utilized differential expression results from a third study, the “Mayo Clinic RNAseq study”, comprised of 82 PSP subjects and 76 control subjects [3], for additional annotation suppl. text (Online Resource 3). These are available to the research community through the AMP-AD knowledge portal (10.7303/syn2580853) and entitled as Mayo RNASeq study (syn6090813). All subjects were North American Caucasians. Study and subject demographics are outlined in Table 1. This study was approved by the Mayo Clinic Institutional Review Board.

**Table 1:** Samples and subject demographics

Cohort	*N*	Females (%)	Age at death (SD)	Mean RIN (SD)
WG-DASL: cohort A	173	77 (45%)	73.38 (7.22)	7.34 (0.83)
WG-DASL: cohort B	95	40 (42%)	71.64 (5.30)	7.04 (1.04)
RNAseq: PSP	82	33 (40%)	73.95 (6.51)	8.48 (0.50)
RNASeq: control	76	38 (50%)	83.72 (9.29)	7.61 (1.04)

Cohorts A and B are independent and composed of PSP subjects with brain expression measurements using WG-DASL microarrays. RNAseq-PSP and RNAseq–control cohorts are from the Mayo Clinic RNAseq study. RNAseq-PSP subjects are a subset of Cohort A. Cohorts A and B are used in the gene expression and transcriptome association analyses. RNAseq-PSP and RNAseq–control data are used in differential gene expression (DGE) analysis

*RIN–RNA* Integrity Number, *N* Number of subjects, *SD* standard deviation, *WG-DASL* Whole Genome cDNA Annealing Selection Extension and Ligation, *RNAseq* RNA sequencing

### RNA isolation

Total RNA was isolated from frozen postmortem brain tissue sampled from the temporal cortex region, for all subjects. For Cohort A [2] and Cohort B [2, 60], RNA was isolated using an Ambion RNAqueous kit according to the manufacturer’s instructions. For the Mayo Clinic RNAseq study [3], RNA was isolated from homogenized tissue using Trizol^®^ reagent followed by DNase treatment and clean-up using Qiagen RNeasy columns. All RNA samples were assessed for quality and quantity using the Agilent 2100 Bioanalyzer and Agilent RNA 6000 Nano Chip and had an RNA integrity number (RIN) ≥ 5.0 (Table 1).

### Gene expression measurements and processing

RNA was transferred to the Mayo Clinic Genome Facility (MGF) core for expression measures; samples were randomized within each cohort prior to transfer. The Illumina Whole Genome DASL (WG-DASL) microarray (Illumina, San Diego, CA, USA) was used to collect transcriptome-wide expression measures for Cohort A and Cohort B as previously described [2, 60]. Cohort A utilized an array with 29,285 probes and Cohort B an array with 24,525 probes, all of which were present in the Cohort A array. For both cohorts, raw probe levels were exported from GenomeStudio software (Illumina Inc.) and the Lumi package (Bioconductor) was used for preprocessing with background subtraction, variance stabilizing transformation, quantile normalization and probe filtering [19]. Probes were screened for the presence of common variants (> 1%) within the probe sequence according to dbSNP138. We limited our analysis to expression probes detected above background in > 50% of subjects in each cohort and that were common to both cohorts, resulting in inclusion of 17,857 probes with identical sequence in both arrays [suppl. Table 27 (Online Resource 1)]. For the Mayo RNASeq cohort, expression measures were collected using RNA sequencing as described previously [2, 3] and detailed elsewhere [suppl. text (Online Resource 3)].

### Genome-wide genotypes

Subjects in Cohort A were previously genotyped using Human 660 W-Quad Infinium BeadChips (Illumina, San Diego, CA, USA) as part of a published PSP risk genome-wide association study [26]. Subjects in Cohort B were previously genotyped using the HumanHap300-Duo Genotyping BeadChips (Illumina, San Diego, CA, USA) [12]. Subjects in the Mayo Clinic RNASeq cohort were genotyped using Omni 2.5 Beadchips (Illumina, San Diego, CA, USA). Genome-wide genotypes were leveraged as part of sample quality control for all cohorts, and for data analysis for the largest Cohort A [suppl.text (Online Resource 3)]. Following QC, genotype imputation was performed for all cohorts: genotypes were imputed to the HRC reference panel (version r1.1) using the Michigan Imputation Server [16]. Prior to imputation, variant position and alleles were linked to the reference panel using McCarthy Group Tools (URL: http://www.well.ox.ac.uk/~wrayner/tools/). Post imputation, variants with a minor allele frequency less than 2% or an imputation r2 < 0.3 were excluded. VCF files from imputation were converted to PLINK [43] formatted files. Imputed genotypes for Cohort A only were used for eQTL and module QTL analysis [suppl.text (Online Resource 3)].

### Neuropathological latent trait measurements

Continuous quantitative neuropathology measures (latent traits) for four tau lesions: neurofibrillary tangles (NFT), oligodendroglial coiled bodies (CB), tufted astrocytes (TA), and tau neuropil threads (TAUTH), and the combined burden of neuropathology (overall), were generated for 848 PSP samples from the Mayo Clinic brain bank, as previously described [2]. Briefly, semi-quantitative counts (none = 0, mild = 1, moderate = 2, severe = 3) were generated by a single neuropathologist (DWD) using CP13 immunostained sections from 19 brain regions affected in PSP, which include: basal nucleus, caudate/ putamen, globus pallidus, hypothalamus, motor cortex, subthalamic nucleus, thalamic fasciculus, ventral thalamus, cerebellar white matter, dentate nucleus, inferior olive, locus coeruleus, medullary tegmentum, midbrain tectum, oculomotor complex, pontine base, pontine tegmentum, red nucleus, and substantia nigra. Counts across all 19 brain regions were used to estimate neuropathological latent traits, using the *R* statistical software “ltm” package [45] (URL: http://www.jstatsoft.org/v17/i05/). All PSP samples assessed in this study were amongst the 848 for which neuropathological latent traits were generated.

### Gene expression analyses

#### Covariate adjusted residuals

Prior to all subsequent analysis, normalized WG-DASL gene expression measures for Cohorts A and B, and the neuropathological latent trait variables, were adjusted for covariates and residuals obtained. A custom function was implemented using *R* statistical software to apply multi-variable linear regression to all tested variables, adjusting for relevant covariates and extracting residuals. Gene expression measures were adjusted for Age, Sex, RNA integrity number (RIN), RINsqAdj [(RIN–RIN_mean_)^2^] and PCR Plate. Neuropathological latent traits were adjusted for Age and Sex. All neuropathology residuals were plotted to confirm that their distributions were approximately normal [suppl. Fig. 1a–b (Online Resource 2)].

#### Gene expression, neuropathological latent trait correlations

Gene expression and neuropathological latent trait residuals were assessed for correlation using the cor.test function (method = pearson), implemented using *R* statistical software, for cohorts A and B separately. Correlation estimates were converted to the Fisher’s Z scale, to enable meta-analysis, which was performed for all probes in common between Cohorts A and B (17,857 probes) using METAL [57], and the resulting beta-coefficients converted back to their corresponding Pearson correlation estimates. Correlation *p* values for each cohort, and the meta-analysis, were adjusted for multiple tests using false discovery rate (Benjamini–Hochberg) [51]. Unique genes nominally associated with each of the latent traits (*p* value < 0.05, consistent direction across both cohorts) were tested for enrichment of gene ontology (GO) biological processes (BP), split by the direction of the correlation estimate (positive or negative), using Metacore (Thompson Reuters) with the full list of unique genes set as the background. Bar plots summarizing the top 10 GO BP were generated in *R* Fig. 2, suppl.Fig. 4 (Online resource 2). Bubble plots, highlighting non-redundant significant GO BP (Bonferroni adjusted *p* value < 0.05) were generated using REVIGO [53], where bubble color indicates enrichment *p* value and bubble size indicates frequency of the GO term in the underlying database queried from the January 2017 Gene Ontology monthly release. Named GO BP in the bubbles plots are based on *p* value (Gene Ontology) and dispensability scores (REVIGO). Bubbles are presented in two-dimensional space reflective of their similarity as determined using the simRel score [46].

#### Gene annotations

To further annotate the genes assessed in this study and explore possible underlying regulatory mechanisms, we linked the results from the expression–neuropathology correlation analysis, to differential expression (DEG) results from the Mayo RNAseq study (PSP vs. Control), and performed a *cis*-eQTL analysis for Cohort A [suppl.text (Online Resource 3)].

### Weighted gene co-expression network analysis (WGCNA)

Weighted gene co-expression network analysis was performed using *R* package WGCNA [34] version 1.41 for Cohorts A and B separately, utilizing normalized expression residuals (see “Covariate adjusted residuals”) as the input variables. A pairwise correlation matrix $A_{n \times n} = (a_{ij} )$, whose element $a_{i,j}$ equals $(0.5 + 0.5*{\text{cor}}(g_{i} ,g_{j} ))^{12}$, that is, a modified Pearson correlation of probe *i* and probe *j* was generated for each cohort. $A_{n \times n}$ was further transformed to a topological overlap matrix (TOM) and hierarchically clustered based on TOM to obtain probe clusters, or modules, where probes within the same module are correlated. This analysis was performed using blockwiseModules function and the key parameters are softpower = 12, networkType = signed, TOMType = signed. For each module, a signed eigengene (first principal component) was computed to represent the overall expression pattern of all probes in that module. Hierarchical clustering of module eigengenes was performed by the “hclust” function in R [Fig. 3 and suppl. Fig. 8 (Online Resource 2)]. Within and between module clustering in Cohort A was visualized in a correlation heatmap [suppl. Fig. 5 (Online Resource 2)] showing pairwise correlations, from WGCNA, for 2000 probes, randomly selected from all expressed probes. Modules identified in Cohort A were assessed for preservation in Cohort B, by “module preservation” function in WGCNA [suppl. Fig. 6 (Online Resource 2)]. The overlap of genes in each module in Cohort A, with modules in Cohort B, was determined and visualized using *R* statistical software [suppl. Fig. 7 (Online Resource 2)].

#### Module eigengene, neuropathological latent trait correlations

Module eigengenes were assessed for correlation with neuropathological latent trait residuals using the same method as described for single probes (“Gene expression, neuropathological latent trait correlations”), and correlation estimates plotted as a heatmap using *R* package ggplot2 [56] [Fig. 3 and suppl. Fig. 8 (Online Resource 2)].

#### Module annotations

To further understand the biological pathways represented by the co-expression modules, and identify key genes or genetic variants that may influence module genes, all modules were annotated for enrichment or presence of: Gene Ontology (GO) biological processes (BP); genes that are enriched in the five primary CNS cell types (neurons, oligodendrocytes, microglia, astrocytes and endothelia); differentially expressed genes (DEG) identified in the Mayo Clinic RNAseq PSP vs. control cohort; chromosome and PSP disease candidate genes [Table 2, suppl. Table 16 (Online Resource 1)]. Gene ontology (GO) enrichment was done using the “GOenrichmentAnalysis” function in WGCNA and biological processes with a Bonferroni adjusted *p* value < 0.05 for a given module considered significant. Modules were assessed for enrichment of the five CNS cell-type genes [suppl. Table 26 (Online Resource 1)] and other features using a one-sided Fisher’s exact test implemented using *R* statistical software. The selection of the CNS cell-type genes is described elsewhere suppl.text (Online Resource 3).

**Table 2:** Co-expression modules associated with tau neuropathology are enriched for biological and disease-relevant features

Module	Trait	Estimate	*p* value	Enrichment		
				Cell type: *p* value	Disease genes *p* value	Chromosome (*p* value)
M2	CB	0.17	2.56E-02	Neuron: 1.30E-134	0.04	Chr 2 (4.07E-03),
	NFT	0.29	1.27E-04			Chr 5 (2.39E-04),
	TA	− 0.06	4.43E-01			Chr 10 (3.84E-02),
	TAUTh	0.20	7.67E-03			Chr 20 (6.17E-04),
	Overall	0.18	1.54E-02			Chr X (1.82E-03)
M3	CB	− 0.06	4.72E-01	NS	NS	
	NFT	− 0.23	2.20E-03			Chr 11 (8.55E-13),
	TA	− 0.09	2.18E-01			Chr 20 (3.58E-02),
	TAUTh	− 0.18	1.99E-02			Chr 21 (2.07E-02)
	Overall	− 0.15	4.39E-02			
M13	CB	− 0.14	6.38E-02	NS	NS	
	NFT	− 0.25	1.10E-03			
	TA	− 0.06	4.49E-01			Chr 7 (1.02E-02)
	TAUTh	− 0.21	5.52E-03			
	Overall	− 0.20	9.55E-03			

Association of co-expression modules with each of the neuropathological traits, annotated for: CNS cell-type enrichment, candidate disease genes in cis with GWAS index SNPs (Hoglinger. G et al Nat Genetics, 2011), and chromosomal location. Additional features of the modules including hub genes, significant DEGs, disease locus genes, transcription factors (TF) and their eQTLs are depicted in suppl. Table 16

The PSP candidate gene set was defined as those genes, for which an expression probe was available, within 100 kb (+/−) of index SNPs identified by the PSP GWAS [26] (rs1411478 near *STX6*, rs7571971 near *EIF2AK3*, rs1768208 near *MOBP*, rs2142991 near *BMS1*, rs11568563 near *SLCO1A2*, rs8070723 and rs242557 near *MAPT*), with the exception of the Chr17q21 locus (rs8070723 and rs242557) where all genes within the ~900 kb inversion (reviewed [42]) were included due to the extent of linkage disequilibrium across this region. The PSP GWAS index SNPs were those that had significant or suggestive PSP risk association in the PSP GWAS [26].

The differentially expressed gene set was defined as DEGs identified in the Mayo RNAseq cohort (PSP vs. Control) with an FDR adjusted *q* value < 0.1. To identify transcription factors (TFs) which reside within co-expression modules of interest, we utilized the transcriptional regulatory network analysis package TReNA [41] suppl.text (Online Resource 3). Bubble plots highlighting significant GO BP (Bonferroni adjusted *p* value < 0.05) for targeted modules were generated using REVIGO [53] as described under the “Gene expression, neuropathological latent trait correlations**”** methods section.

#### Network plots

Network plots were generated for the WGCNA outputs using Cytoscape v3.2.0 (http://www.cytoscape.org/). For each module of interest, unique genes were identified; where more than one probe or transcript accounted for the same gene, and only the one with the highest module membership (MM) was retained. Module genes with a MM ≥ 0.7 were extracted from module-specific topological overlap matrices (TOM) generated by WGCNA. Probe pairs were sorted according to TOM weight and the top 150 pairs according to TOM weight (range 0–0.31) were imported into Cytoscape, along with the following additional characteristics for each gene: the most significant neuropathological latent trait associated with transcript (*p *< 0.05) and whether the gene is enriched in a specific CNS cell-type. The Cytoscape Network Analysis tool was used to calculate degree (number of connections each node has in the network), using continuous scale mapping ranging from the minimum degree of nodes to the maximum degree of nodes in the network. Latent trait association and CNS cell-type enrichment for the transcripts plotted in the networks were used to apply visual mapping styles as indicated in Fig. 4. Networks were arranged according to Cytoscape’s prefuse force directed by edge betweeness layout algorithm.

#### Venn diagrams

**Fig. 1:**
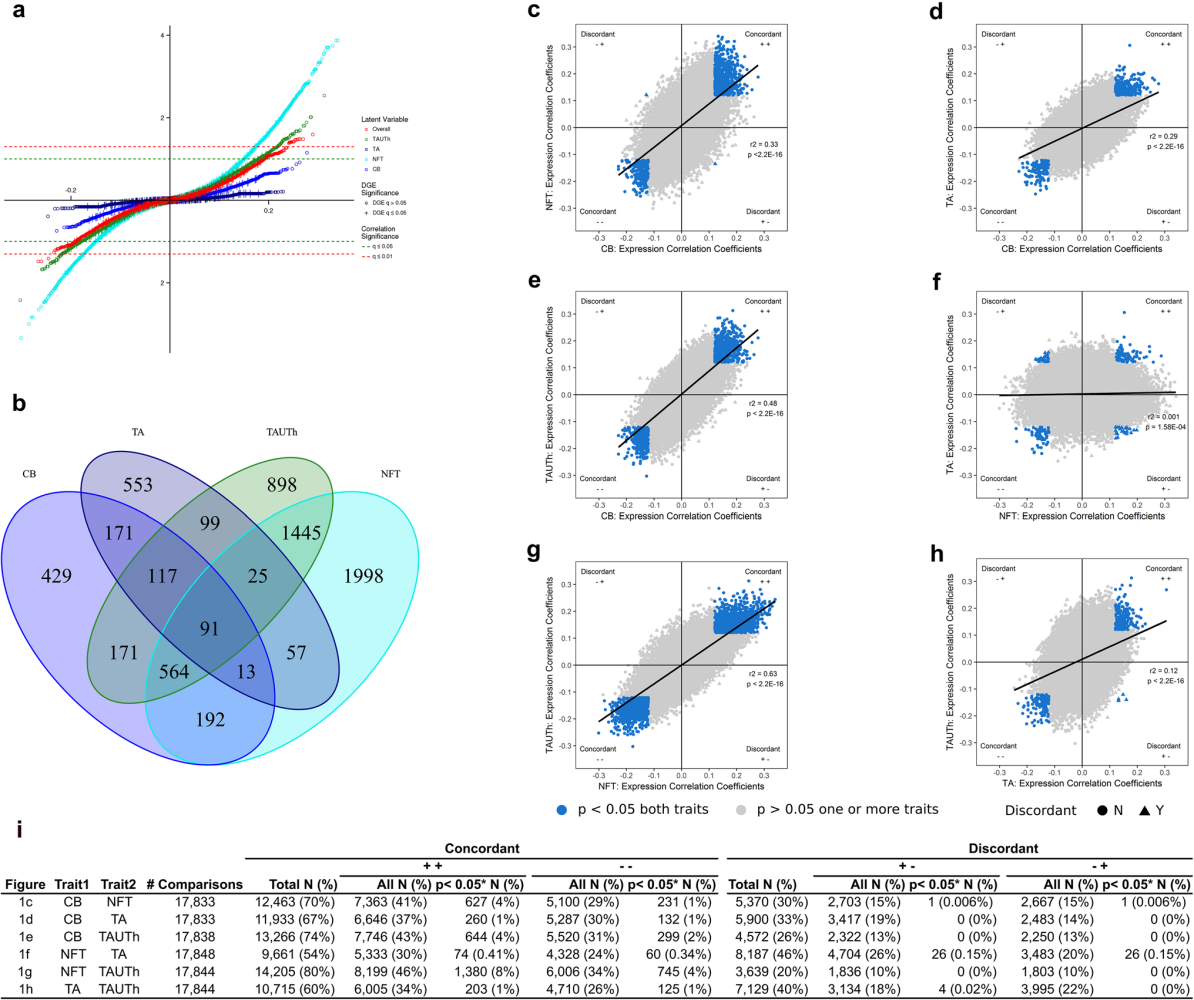
Temporal cortex gene expression levels are associated with tau neuropathology. Meta-analysis results from Cohorts A and B are presented for genes expressed in the temporal cortex tissue of PSP patients. Results for each of the latent neuropathology traits, i.e., Overall, Tau Threads (TAUTh), Tufted Astrocytes (TA), Neurofibrillary Tangles (NFT) and Coiled Bodies (CB) are shown in a scatter plot **a** where the X-axis represents the Pearson correlation coefficient and the Y-axis represents the *q* value for significance. Two significance thresholds are indicated by green (*q* < 0.05) and red (*q* < 0.01) lines. Genes that are also differentially expressed between PSP patient vs. control temporal cortex samples are indicated with a “+” and those that are not, with an open circle. **b** Venn diagram representing overlap in nominal associations (unadjusted *p* value < 0.05) between each of the four tau neuropathology traits. **c**–**h** Scatter plots comparing the Pearson correlation coefficients for probe-latent trait associations, between each pair of traits, CB vs. NFT **c**, CB vs. TA **d**, CB vs. TAUTh **e**, NFT vs. TA **f**, NFT vs. TAUTh **g**, and TA vs. TAUTh **h**. The X-axis indicates the correlation coefficient for the first trait and the Y-axis the correlation coefficient for the second trait. Probes that are nominally significant (unadjusted *p* value < 0.05) for both traits are highlighted in blue. Probes that are concordant in direction of association for both traits are indicated as circles, and discordant probes are indicated as triangles. The direction of association with reference to the trait on the X-axis, followed by the trait on the Y-axis is indicated in each quadrant of each plot (− negative transcript-trait associations; + positive transcript-trait associations). The Pearson correlation *r*2 and *p* value for the overall comparisons of transcript associations for each pair of traits are shown on the plots. **i** Table summarizing the number of concordant and discordant probes for each pair of traits, split by direction of the correlation coefficient (negative or positive). Between 9 and 24 probes have a correlation coefficient of zero for a given pair of traits and are hence not counted. *columns indicate count for number of probes with a *p* value < 0.05 (unadjusted) for both traits

Probes that were nominally associated (unadjusted *p* < 0.05) in the meta-analysis of Cohorts A and B, with each of the four neuropathological latent variables, were identified. Venn diagrams illustrating the overlapping probes and genes, with respect to the direction of the association, were generated using *R* package VennDiagram (Fig. 1).

#### Circos plot

The circos plot was made using *R* packages Circulize. The outermost track is the ideogram of human reference genome hg19. The second outermost track shows a barplot of − log10(DEG p value) for genes with DEG *p* value < 5E-03. The next panel contains five tracks of barplots, which are from outermost to innermost, the − log10 of association p value between gene expression and latent variables Overall, TAUTh, TA, NFT, CB. The next panel contains three tracks. From outermost to inner most, they indicate the top 150 genes that are in the highlighted modules CohortA_M13, CohortA_M3 and CohortA_M2, respectively, according to module membership.

### Protein studies

#### Proteome analysis

Temporal cortex samples from the Mayo Clinic RNAseq study underwent proteome measurements using Liquid Chromatography Coupled to Tandem Mass Spectrometry (LC–MS/MS) analysis. These data were downloaded from syn9637748, and the Synapse ID of the file containing the mapping of protein ID and TCX ID is syn9782771. Methodological details are available from synapse and previously published [5, 13]. We obtained LFQ (Label-Free Quantification) intensities for 82 PSP samples and log2 transformed these measures. Peptides with median log2(LFQ) levels of at least four were kept. The combat function in *R* sva package was applied to adjust the batch effect using non-parametric approach. The combat method took batch information, sex and age at death to estimate batch-specific location and scale adjustment of the log2 transformed LFQ intensities, and returned batch-adjusted values. There are 6585 measured peptides encoded by 5846 genes. Out of these 5846 genes, 5148 are also present in WGCNA analysis which encompass 18,516 genes in total, 919 are present in module 2 (2282 genes), 219 present in module 3 (2347 genes), and 161 present in module 13 (701 genes). There are 4022 unique peptides (4016 genes) that have median log2(1 + LFQ) >  = 4, false discovery rate below 1% and without contamination. Out of these 4016 genes, 3627 are present in WGCNA analysis, 714 in module 2, 97 in module 3 and 100 in module 13. For these 4022 peptide pass-QC (4016 genes), residuals were generated for each peptide by linear regression where the dependent variable was the batch-adjusted values from combat and independent variables were sex and age at death. Out of these 82 samples, 76 are also in Cohort A. Pearson correlation and *p* value between protein level residuals of these 76 samples and five latent traits were calculated using *R* cor and cor.test function.

#### Immunohistochemistry

We selected NSF, SLC1A4 and MAP4 for our immunohistochemistry studies. Besides availability of quality antibodies, our selections are based on potential biological relevance of these proteins to PSP. *NSF* resides in a PSP risk-associated region on chromosome 17 [8] and plays a key role in synaptic vesicle release [44]. *SLC1A4* encodes a brain amino acid transporter, mutations of which lead to severe intellectual disability, microcephaly and spasticity [25, 49]. *MAP4* encodes a microtubule-associated protein and is evolutionarily linked to MAPT [52]. Immunohistochemistry was performed on ten PSP patients (five male, five female) with transcriptome and latent trait data. Paraffin-embedded 5-μm thick sections from the Putamen, Amygdala, Globus pallidus and Basal nucleus, mounted on glass slides were immunostained using a DAKO Autostainer (Dako, Carpinteria, CA) as previously described [30]. These regions were selected to represent a range of pathologically affected regions (suppl. Fig. 2c). Primary antibodies against MAP4 (rabbit polyclonal; 1:250; 11,229-1-AP; ProteinTech, Chicago, IL), SLC1A4 (rabbit polyclonal; 1:250; ab118454; Abcam, Cambridge, MA), and NSF (rabbit polyclonal; 1:500; PA5-76,126; Thermo Fisher Scientific, Carlsbad, CA) were used. Following deparaffinization in xylene and reagent alcohol, antigen retrieval was performed by steaming slides in a Tris/EDTA buffer, pH 9 (Dako) for MAP4 and NSF or a Citrate buffer, pH 6 (Dako) for SLC1A4 for 30 min. Sections of basal ganglia were processed for double-labeling immunohistochemistry with the combination of anti-tau (CP13, 1:1000) and anti-MAP4 antibodies (1:250).

## Results

### Human brain gene expression levels associate with PSP tau neuropathology

Brain expression levels from temporal cortex, which is an area relatively unaffected by PSP pathology, were measured using expression microarrays in two independent Cohorts “A” and “B”, collectively comprised of 268 autopsied PSP patients (Table 1). All patients had continuous quantitative latent trait phenotypes for the four types of PSP tau neuropathology (CB, NFT, TA, TAUTh, Overall) [2, 24] [suppl. Fig. 1a–b (Online Resource 2), Methods, suppl. text (Online Resource 3)]. All latent neuropathology traits had positive pairwise correlations with one another in both cohorts, to varying degrees [suppl. Fig. 2a–b (Online Resource 2), suppl. text (Online Resource 3)]. The strongest correlation was observed between NFT and TAUTh and weakest between NFT and TA.

To identify individual gene expression changes associated with tau neuropathology in PSP, we initially focused on meta-analysis results from Cohorts A and B that passed the stringent Bonferroni cutoff meta-analysis *p* value < 2.80E-06, after adjusting for the 17,857 expression probes which were common to both cohorts. This is an overly conservative correction, given the presence of multiple expression probes for some genes and correlated expression levels for some probes. Despite this, we identified 43 probes (41 unique genes) with expression–neuropathology associations significant after Bonferroni correction [suppl. Table 1 (Online Resource 1)]. Most of these associations were with the NFT neuropathology (37 probes/36 unique genes), and only 4 with TAUTh, 1 with TA and none for CB.

We postulate that gene expression levels that associate uniquely with one tau neuropathology may underlie a specific aspect of disease pathophysiology. In contrast, genes associated with all four neuropathology traits may represent genes involved more generally in tau neuropathology. Of the genes most significantly associated with tau neuropathology [suppl. Table 1 (Online Resource 1)], there was no overlap between the different traits, except for *CCDC28A* that was associated with both TAUTh and Overall neuropathology. To further assess the extent of overlap, we focused on the more sizable nominally significant associations at unadjusted meta-analysis *p *< 0.05. There was considerable enrichment of nominally significant associations for NFT (25%), TAUTh (19%) and CB (10%), but not for TA (6%) [Fig. 1a, suppl. Tables 2–6 (Online Resource 1)]. This suggests that TA may be least influenced by brain gene expression changes, in comparison to the other latent traits. The majority of these nominal associations were positive (64% NFT, 61% TAUTh, 68% CB, 59% TA), indicating that higher temporal cortex expression levels for most of these genes associated with greater burden of tau neuropathology. Of the nominally significant expression–neuropathology associations, considering each trait individually, nearly half were unique to NFT (46%) or TA (49%), and about a quarter unique to TAUTh (26%) or CB (25%) (Fig. 1b). This may suggest that NFT and TA may have the greatest extent of distinct expression associations. We further classified overlapping nominally significant genes for each pairwise comparison as “concordant” or “discordant” with respect to the direction of the correlation coefficients (Fig. 1c–i**)**. The greatest concordant overlap was observed for genes associated with CB, NFT and TAUTh (NFT–TAUTh = 12% of all tested probes; CB–TAUTh = 6%; CB–NFT = 5%) and the least for comparisons with TA (CB–TA = 2%; TA–TAUTh = 2%; NFT–TA = 0.75%). This is further supported by the overall correlations between neuropathology association coefficients for each pair of traits (Fig. 1c–h). These pairwise analyses suggest that brain expression changes most commonly influence NFT and TAUTh, whereas NFT and TA are mostly influenced by distinct expression changes. Very few nominally significant genes were discordantly associated (Fig. 1i), with the exception of NFT–TA, where 52 transcripts (0.3% of all tested) had discordant direction of associations with these two traits, again consistent with their divergent patterns of expression associations. These findings suggest that astrocyte neuropathology (TA) may be most distinct from other neuropathologies in PSP by both the extent and type of gene expression associations. Further, brain expression changes may have the strongest influence on neuronal PSP pathology (NFT), followed by TAUTh and oligodendroglial CB traits.

We sought to determine which of the most significant transcriptional associations we observed [suppl. Table 1 (Online Resource 1)] were likewise reflected at the protein level. For a subset of the Cohort A samples (*n *= 76), proteome data was also available [suppl. Table 1 (Online Resource 1)]. Of the 41 genes with the most significant expression–neuropathology associations, 20 had proteome data. Despite the smaller cohort, two of the proteins had nominally significant associations in the same direction as that for gene expression, including the most significantly associated *GABRB3*.

There were only 91 probes (90 unique genes) that associated with all four neuropathologies at nominal significance. All of these had a consistent direction of association with all neuropathologies. Although some of these genes have been implicated in tau biology [27], cognition [37] or AD [22], the functional implications for most of these “common tau neuropathology” associated genes remains to be established [suppl. Table 7 (Online Resource 1), suppl. text (Online Resource 3)].

A PSP risk GWAS identified common genetic variants associated with disease risk [26]. We hypothesized that some of these variants may influence disease risk by regulating brain expression levels of nearby gene(s), which in turn modify neuropathology. To determine whether any of the genes at the PSP GWAS risk loci [26] have brain levels that associate with tau neuropathology in PSP, we assessed all genes that are within +/− 100 kb of the PSP risk association variant in addition to the 900 kb inversion region on chromosome 17q21 [suppl. Table 8 (Online Resource 1), suppl. text (Online Resource 3)]. After correcting for 39 tested transcripts at PSP risk loci, 6 were significantly associated with neuropathology. Of these transcripts, five were from three genes (*MAPT, NSF, CRHR1*) located at the chromosome 17q21 inversion region associated with risk of PSP and other neurodegenerative conditions [4, 31, 47]. All but one of these chromosome 17 expression probes (ILMN_1800049: MAPT, Exon 6) is upregulated in samples with more NFT pathology. Additionally, brain *NSF* levels are also *positively* associated with TAUTh and Overall traits; and *IAPP* on chromosome 12 has *negative* association with NFT. Many of the other genes at the PSP risk loci were also nominally significant, though they did not reach study-wide significance (*ARL17B, C2ORF51, IER5, KIAA1267, LRRC37A2, MOBP, MR1, SLCO1A2, STX6*).

Given its strong association with PSP neuropathology in our study, its proximity to the chromosome 17 inversion region [8] and its location within a region of copy number polymorphisms [50], we investigated the exon levels of NSF using the RNAseq data from 82 PSP subjects [3]. We determined that exons 1–2 and 10–21 were expressed (NM_006178) in the temporal cortex [suppl. Fig. 3 (Online Resource 2)]. Although there was evidence of variability in the levels of the exons, no strong NSF exon *cis*-eQTLs were identified [suppl. Table 9 (Online Resource 1)], suggesting that the NSF expression–neuropathology associations are unlikely to be driven by *cis*-regulatory variants.

#### Biological pathways are enriched for genes with PSP neuropathology associations

**Fig. 2:**
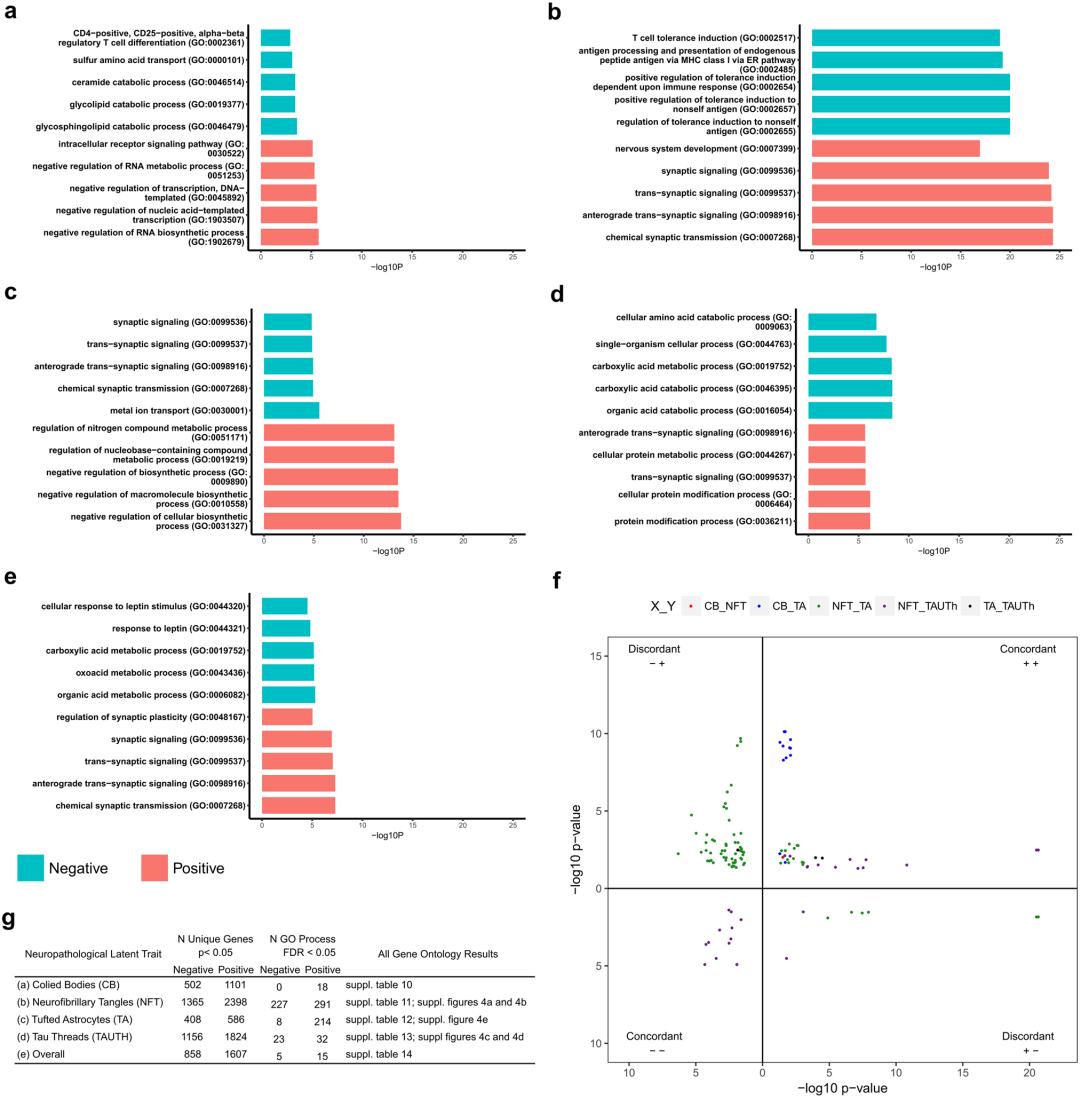
Temporal cortex gene expression levels associated with tau neuropathology are enriched for Gene Ontology biological processes. **a**–**e** Gene Ontology bar plots for the five most significant GO terms for genes nominally associated with CB **a**, NFT **b**, TA **c**, TAUTh **d** and Overall **e**. The X-axis indicates the unadjusted GO term enrichment *p* value. Note that all GO terms shown in **a**–**e** are significantly enriched at FDR < 0.05, except those for CB negative associations. **f** Scatter plot for GO terms (FDR < 0.05) common to two or more traits. Each point on the plot represents a single GO term, the position on the plot is determined by the enrichment *p* value on the X and Y-axes for trait 1 and trait 2, respectively, and as indicated in the key. Each quadrant represents one of the four combinations of direction of associations for each pair of traits with the GO-term-enriched transcripts. Upper right and lower left quadrants include the concordant positive and negative associations for trait pairs. Upper left and lower right quadrants represent discordant associations, in the negative–positive and positive–negative directions, respectively. The first direction pertains to trait 1 and the second direction to trait 2 in all quadrants. **g T**able summarizing the number of nominally associated unique genes with each of the neuropathology traits. The number of GO terms that are significant at false discovery rate adjusted *p* value < 0.05 is also shown, split by direction of the correlation coefficient (negative or positive)

To determine if any biological pathways are enriched for genes with nominally significant brain expression–neuropathology associations, we used Metacore software to identify enriched Gene Ontology (GO) terms [1, 7]. Gene sets associated with each neuropathological trait in a specific direction (positive or negative) were tested separately for GO term enrichment. We identified significant GO terms (FDR < 0.05) for all gene sets, with the exception of genes negatively associated with CB [suppl. Tables 10–14 (Online Resource 1)]. The five most significantly enriched GO terms for each tested gene set are shown in Fig. 2a–e. There were more significant GO terms for genes positively associated with neuropathology (Fig. 2g). This may be expected given that there are more genes positively associated with all of the neuropathology traits. However, the number of GO terms enriched for positively associated genes was disproportionately higher for CB (100% of all associated genes) and TA (96% of all associated genes).

The largest numbers of significantly enriched GO processes were for the NFT-associated genes (*n *= 518, 56% with positive associations); followed by TA (*n *= 222), then TAUTh (*n *= 55, 58% with positive associations), and CB (*n *= 18). To better visualize the large number of GO processes, we generated REVIGO [53] plots for those gene sets with > 20 significantly enriched GO processes [suppl. Fig. 4a–e (Online Resource 2)]. This approach identified clusters of GO processes with distinct patterns of enrichment for the different neuropathologic trait associations. Genes *positively* associated with NFT were most significantly enriched for “synaptic” GO processes, whereas “immune system” terms were the most significant for *negative* NFT-associated genes [Fig. 2b, suppl. Fig. 4a–b (Online Resource 2), suppl. Table 11 (Online Resource 1)]. In complete contrast, “synaptic” GO terms were enriched for genes with *negative* TA-expression associations, whereas “immune system” terms were enriched for *positively* TA-associated genes [Fig. 2c, suppl. Fig. 4e (Online Resource 2), suppl. Table 12 (Online Resource 1)].

“Synaptic” GO processes were also the most significantly enriched for *positive* TAUTh-associated genes, in addition to “protein modification” terms [Fig. 2d, suppl. Fig. 4d (Online Resource 2), suppl. Table 13 (Online Resource 1)]. Another consistency was for “RNA metabolism” GO terms that were enriched for genes *positively* associated with both TA and CB [Fig. 2a, suppl. Table 10 (Online Resource 1)]. GO enrichment for genes associated with the Overall neuropathology trait [Fig. 2e, suppl. Table 14 (Online Resource 1)] generally reflected the most significant *positive* NFT associations (“synaptic”), and *negative* TAUTh-associations (“metabolic processes”) [suppl. Fig. 4c (Online Resource 1)]. We assessed overlapping GO terms for each pair of traits, and similar to the probe level analysis, identified the most concordance for TAUTh and NFT-associated GO terms, and the least for NFT and TA-associated GO terms (Fig. 2f).

### Co-expression modules associate with PSP neuropathology

**Fig. 3:**
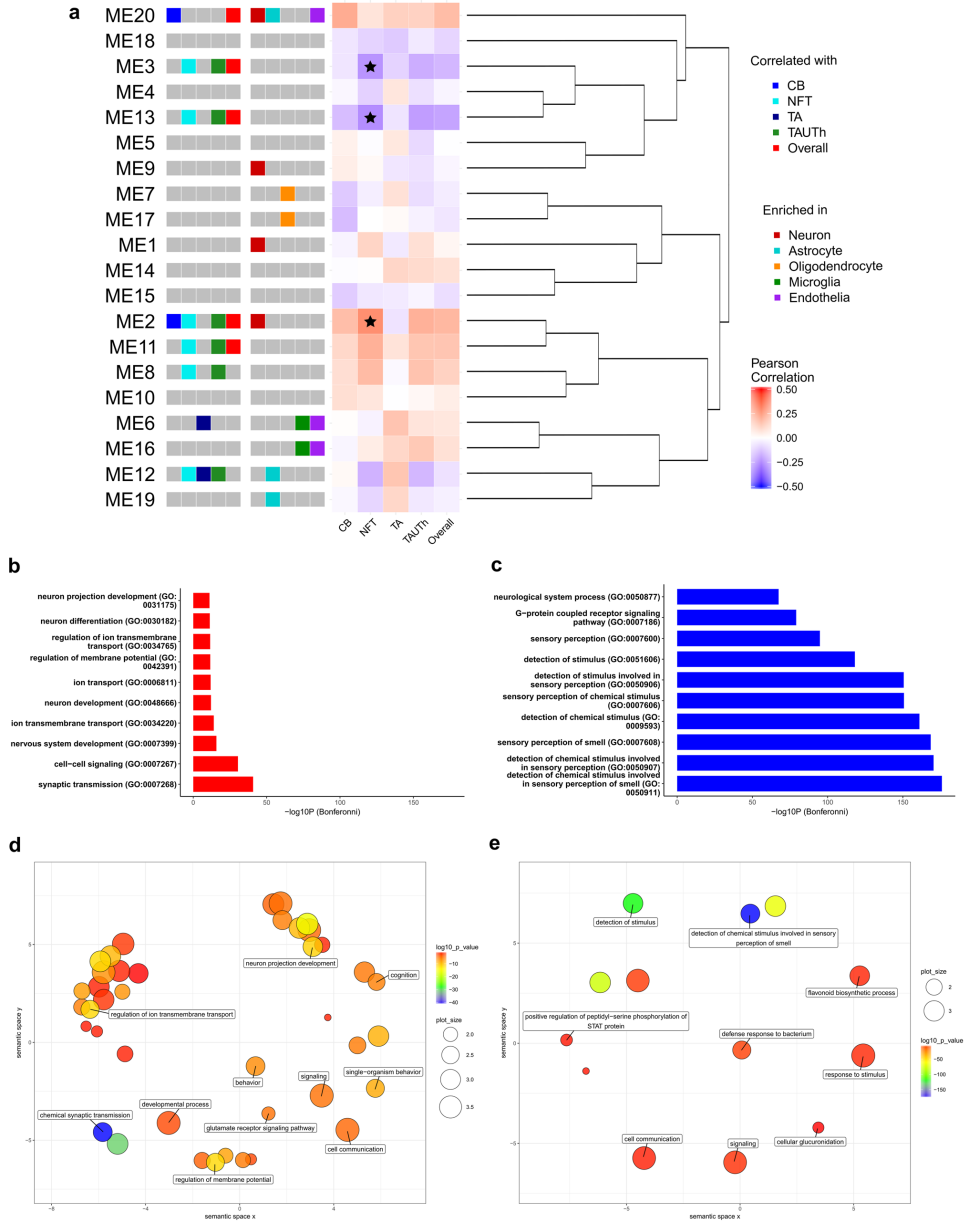
Co-expression modules are associated with tau neuropathology and enriched for cell type marker genes and Gene Ontology biological processes. Correlation and enrichment results are presented for co-expression modules and module eigengenes. **a** Left to right, neuropathology barcode indicating module eigengenes (MEs) nominally associated (unadjusted *p *< 0.05) with CB, NFT, TA, TAUTh or Overall according to the legend in the top right. Cell type enrichment barcode indicating modules enriched for genes that are predominantly expressed in CNS cell types, color coded according to the middle right legend. Heatmap illustrating the Pearson correlation for each ME with the neuropathological traits, where Bonferroni-significant MEs are indicated with a star. Hierarchical clustering dendrogram depicting the relationship between MEs. **b**–**c** Gene Ontology bar plots indicating the ten most significant GO terms for modules 2 **b** and 3 **c**. These are the Bonferroni-significant modules that also had significant GO term enrichment. **d**–**e** REVIGO scatter plots clustering significant (FDR < 0.05) GO terms for module 2 **d** and 3 **e** in two-dimensional space according to semantic similarities. Bubble color reflects the enrichment *p* value; the size of the bubble refers to the size of the GO term; labeled bubbles are the most unique terms in the cluster

Analyses of individual gene expression levels strongly suggest the presence of clusters of genes with either distinct or overlapping patterns of association with PSP neuropathology traits. To formally identify groups of co-expressed genes and their patterns of tau neuropathology associations, we performed Weighted Gene Co-expression Network Analysis (WGCNA) [33, 34]. Twenty co-expression modules were identified in Cohort A [Fig. 3a, suppl. Table 15 (Online Resource 1)], eight of which are associated with at least one neuropathology trait (unadjusted *p *< 0.05). Of these eight modules, three remain significant even after performing a Bonferroni correction for 20 tests (*p *< 2.5E-3); CohortA_M2, CohortA_M3 and CohortA_M13 modules [Table 2, suppl. Table 16 (Online Resource 1)].

Similar to the individual gene findings, NFT had the greatest number and most significant associations with co-expression modules. Six of the eight modules are associated with NFT (unadjusted *p *< 0.05), three in a positive (CohortA_M2, CohortA_M8 and CohortA_M11) and three in a negative direction (CohortA_M3, CohortA_M12, CohortA_M13). TAUTh was likewise associated with the same six modules, and in the same direction as NFT, but with slightly weaker effect sizes. CB was positively associated with two modules (CohortA_M2, CohortA_M20), one of which also positively associated with NFT and TAUTh.

In contrast, TA had a divergent pattern of associations in comparison to the other neuropathology traits. Two modules were *positively* associated with TA, one of which was not associated with any of the other neuropathologies (CohortA_M6). The other (CohortA_M12) was *negatively* associated with both NFT and TAUTh, further highlighting the divergence of brain gene expression associations between TA and the other tau pathologies.

**Fig. 4:**
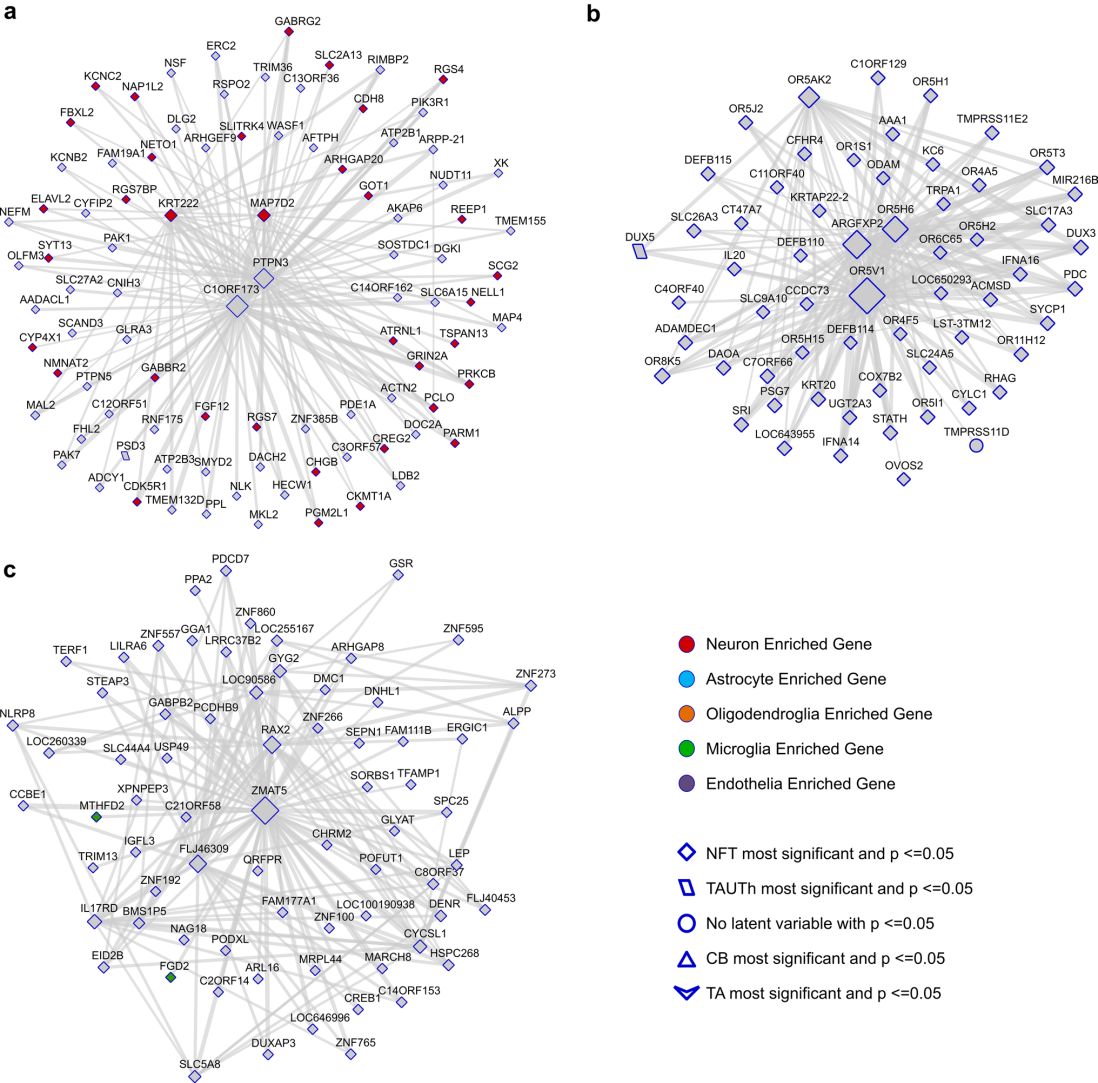
Network plots for Cohort A co-expression modules 2, 3 and 13. Genes that have a module membership (MM) > 0.7 and are amongst the top 150 connections, based on their degree of correlation in the TOM matrix, are shown for CohortA_M2 **a**, CohortA_M3 **b** and CohortA_M13 **c**. The size of the nodes reflects the number of connections (edges) and the weight of the edges reflects the strength of correlation between two nodes. Nodes are shaped according to the most significant neuropathological trait (bottom right legend) and colored according to whether they are predominantly expressed in one CNS cell type (top right legend)

The most significant module was CohortA_M2, which associated *positively* with NFT (*p *= 1.27E-4), and nominally (*p *< 0.05) with TAUTh, CB and Overall traits, but not with TA [Figs. 3a, 4a, Table 2, suppl. Table 16 (Online Resource 1)]. This module was enriched for neuronal genes and interestingly included 33 of the 41 genes with the most significant neuropathology associations [suppl. Table 1 (Online Resource 1)]. The other two significant modules, CohortA_M3 and CohortA_M13 are clustered closely on the module dendrogram, and are not significantly enriched for any CNS cell type. Both CohortA_M3 and CohortA_M13 have significant *negative* associations with NFT, and nominally associate with TAUTh and Overall traits [Figs. 3a, 4b–c, Table 2, suppl. Table 2 (Online Resource 1)]. Given these findings and the high degree of correlation between genes in CohortA_M3 and CohortA_M13, these two modules are likely components of the same broader co-expression network [suppl. Fig 5 (Online Resource 2)].

We sought replication of the Bonferroni-significant Cohort_A module results in Cohort_B, which had 28 co-expression modules [suppl. Table 17 (Online Resource 1)]. The significant modules CohortA_M2, CohortA_M3, and CohortA_M13 are well preserved in Cohort B [suppl. Fig. 6 (Online Resource 2)], where CohortA_M2 has the highest level of preservation amongst all modules. The Cohort B modules with the highest number of overlapping transcripts with the significant Cohort A modules are as follows: Cohort B_M1 for Cohort A_M2 and CohortB_M5 for both CohortA_M3, and CohortA_M13 [suppl. Fig. 7 (Online Resource 2)]. CohortB_M1 is enriched for neuronal and to a lesser extent also astrocyte gene signatures [suppl. Fig. 8 (Online Resource 2), suppl. Table 17 (Online Resource 1)], and such as Cohort A_M2, is positively associated with NFT (*p *= 4.19E-02). The correlation coefficients for NFT associations with CohortA_M2 (*r *= 0.29) and CohortB_M1 (*r *= 0.21) are remarkably similar. CohortB_M5 has no CNS cell enrichment, similar to CohortA_M3 and CohortA_M13. Although CohortB_M5 does not have significant associations with neuropathology traits, it had a negative trend of association with NFT (*r *=  − 0.14, *p *= 0.19), consistent with its Cohort A counterparts.

### Biological characteristics of co-expression modules have divergent features for distinct PSP neuropathology traits

To further explore the biological features of the neuropathology-associated co-expression modules, we tested for enrichment of the module genes in GO terms [Fig. 3b–e, suppl. Tables 18–21 (Online Resource 1)]. For CohortA_M2, we identified enrichment for 81 significant GO biological processes (BP) after Bonferroni adjustment, where “synaptic” terms were the most significantly enriched [Fig. 3b, d, suppl. Table 18 (Online Resource 1)]. Consistently, “synaptic” GO terms were also significantly enriched for CohortB_M1 genes [suppl. Table 19 (Online Resource 1)]. CohortA_M3, which is negatively associated with NFT and TAUTh, is enriched for GO terms related with smell sensation (Fig. 3c, e). CohortA_M13 does not have any GO term enrichment. CohortB_M5 is also enriched for “olfactory” terms, consistent with CohortA_M3. Thus, the most significant modules in Cohort A have consistent GO term enrichment with their Cohort B counterparts.

To delineate the biological features for the co-expression modules that have divergent associations with TA vs. other neuropathologies, we next focused on CohortA_M6 and CohortA_M12. Interestingly, CohortA_M6, which is *positively* associated with TA, is enriched for “immune system” terms [suppl. Table 18 (Online Resource 1), suppl. text (Online Resource 3)], consistent with individual gene expression associations for this neuropathology trait [suppl. Fig. 4e (Online Resource 2)]. CohortA_M12, which is *positively* associated with TA, but *negatively* with NFT and TAUTh, is enriched for “metabolic processes” terms, again reflecting the individual gene expression–neuropathology associations [Fig. 2a–e, suppl. Fig. 4a, c, e (Online Resource 2)].

We further annotated the most significant modules [Table 2, suppl. Table 16 (Online Resource 1)] for enrichment of PSP candidate risk genes, defined as +/− 100 kb of the PSP risk association variant [26] in addition to the 900 kb inversion region on chromosome 17q21. CohortA_M2 was the only module significantly enriched for PSP candidate risk genes, which includes chromosome 17q21 inversion region genes *MAPT* (probe ILMN_2310814), *NSF* and *CRHR1*, which are also individually associated with neuropathology traits [suppl. Table 8 (Online Resource 1)], in addition to *IER5* and STX6 on chromosome 1; and *BMS1* on chromosome 10.

### Genomic annotations of co-expression modules

To determine whether the expression levels in the most significant co-expression modules [Table 2, suppl. Table 16 (Online Resource 1)] may be driven by nearby expression quantitative trait loci (*cis*-eQTL), we tested whether the individual genes in these modules had significant brain *cis*-eQTL [suppl. text (Online Resource 3), suppl. Table 22 (Online Resource 1)]. Many of the genes had significant eQTL, *PRSS36* in module 2, *C19ORF48* in module 3 and *ZC3H12D* in module 13 had variants that remained significant after conservative correction for the number of tests [suppl. Table 22 (Online Resource 1)]. We also ran eQTL analysis for the module eigengenes that represent modules 2, 3 and 13 [suppl. Tables 23–25 (Online Resource 1), suppl. Fig. 9a–c (Online Resource 2)], suppl.text (Online Resource 3) and generated false discovery rate (Benjamini–Hochberg) adjusted *q* values. This analysis identified 53 SNPs at 11 loci on 6 chromosomes (Chr1, Chr4, Chr5, Chr7, Chr8, Chr17) associated with ME2, 8 SNPs at 5 loci on 3 chromosomes (Chr3, Chr19, Chr20) associated with ME3, and 2 SNPs at 2 loci on Chr 4 associated with ME13, with a *q* value < 0.05. The most significant locus for ME2 [suppl.Table 23 (Online resource 1)] includes 14 SNPS (*q *< 0.05) that reside within an uncharacterized ncRNA on Chr1; for ME3 the most significant SNP (rs115709231) is located within an intron of *HDAC11*; for ME13 the most significant SNP (rs111571429) is located within an intron of *GABRB1*. *HDAC11* and *GABRB1* are not members of Modules 3 or 13, respectively, so this association is unlikely to be an artifact of a *cis*-eQTL association with these genes. Given that these variants are imputed, additional studies are required to validate and replicate these observations.

**Fig. 5:**
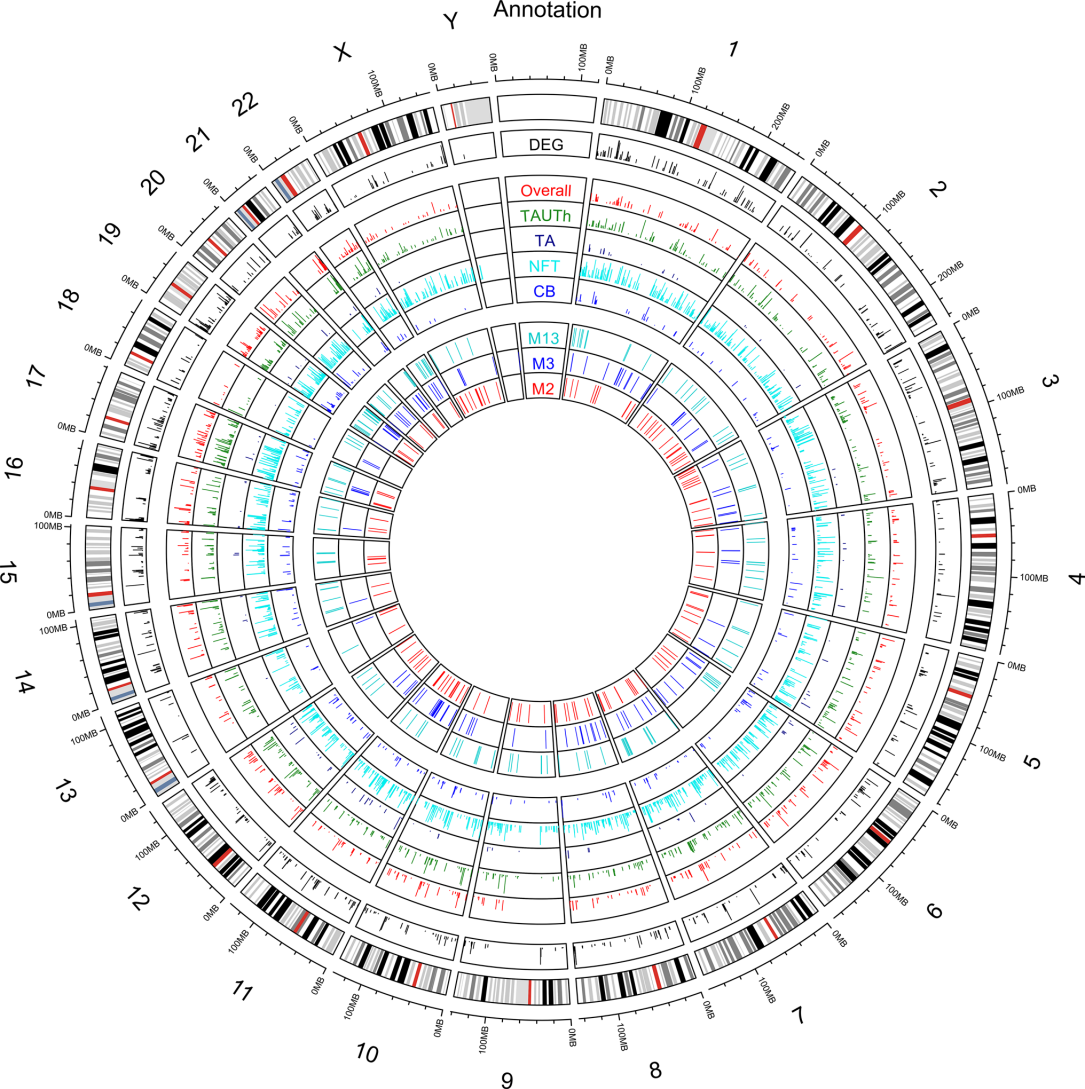
Circos plots of chromosomal locations for tau neuropathology associations. The outermost track is the ideogram of human reference genome hg19. The second outermost track shows a bar plot of − log10(*p* value) for genes that are differentially expressed between PSP and controls. This is limited to genes with DEG *p* value < 5E-03. The next panel contains five tracks of bar plots, which are the − log10 *p* value of association between gene expression and the following latent variables from outermost to innermost: Overall, TAUTh, TA, NFT, CB. The next panel contains 3 tracks, which indicate the 150 genes with the highest module membership from each of the neuropathology-associated modules, CohortA_M13, CohortA_M3 and CohortA_M2, from outermost to innermost, respectively

Given that regulatory elements and genes regulated by these elements can reside in distinct chromosomal domains [23, 28], we next tested the neuropathology-associated modules for overrepresentation of genes located on distinct chromosomes. Indeed, there was enrichment of genes located on certain chromosomes for all three modules [Table 2, suppl. Table 16 (Online Resource 1), Fig. 5]. We also performed transcription factor (TF) regulatory network analysis using TReNA [41] and identified TFs within the three most significant modules, some of which have nominally significant *cis*-eQTL [suppl. Table 16 (Online Resource 1)]. Most of these TFs were highly connected with the other genes in these modules with module membership (MM) values > 0.70. These findings suggest biological co-regulation of genes residing in these co-expression networks.

### Immunohistochemistry studies

We further explored the cellular localizations of select proteins by immunohistochemistry studies. We selected three proteins to represent an NFT-associated gene (*NSF*), a TAUTh-associated gene (*SLC1A4*) and a hub gene from the neuronally enriched module CohortA_M2 (*MAP4*), which is associated with NFT, TAUTh and CB [suppl. text (Online Resource 3)]. Immunohistochemistry patterns of these proteins were consistent with their neuropathology associations [suppl. Fig. 10 (Online Resource 2)].

## Discussion

In this transcriptome-wide association study of brain gene expression levels and quantitative neuropathology measures in PSP, we identified transcripts and expression networks that have unique patterns of association with the distinct cell-specific tau lesions in this disease. Tau pathology in PSP is characterized by neuronal (NFT), astrocytic (TA), oligodendroglial (CB) and white matter (TAUTh) lesions. We took advantage of this diverse cell-specific neuropathology to identify brain transcriptional networks enriched for specific biological processes that may influence different aspects of this disease in a unique fashion. Our findings reveal that neuronal NFT and astrocytic TA pathologies in PSP have the most divergent patterns of brain expression associations. We implicate changes in synaptic, immune and olfactory mechanisms in various aspects of PSP neuropathology, and annotate transcriptional networks for many genomic features, including enrichment of candidate PSP risk variants, chromosomal regions and TFs. Our study design is unique in its use of brain expression measures from the temporal cortex, a relatively unaffected region in PSP [17], from two independent cohorts of 268 autopsied PSP patients, who also had detailed quantitative neuropathology measures. This design enabled us to minimize the potential confounding effects on gene expression due to cell population changes, which occur in diseased tissue [48]. As such our conclusions are unlikely to be driven by gene expression changes that occur as a consequence of neuropathology and rather reflect the influence of upstream regulatory changes on cell-specific tau pathology.

We detected the greatest number and the most significant expression associations for neuronal NFT pathology, at both gene [suppl. Table 1 (Online Resource 1)] and co-expression network levels [Table 2, Figs. 3, 4, suppl. Table 15 (Online Resource 1)]. This is not surprising given that PSP is a primary neurodegenerative tauopathy. Amongst the most significant NFT-associated transcripts are those implicated in tau pathophysiology, including *CDK5R1*, which encodes the regulatory p35 subunit of cyclin-dependent kinase 5, the truncated form (p25) of which hyperphosphorylates tau and is implicated in neuronal death in neurodegenerative diseases [15, 40]. *CDK5R1* is also one of the most well-connected genes in the neuronal modules (CohortA_M2 and CohortB_M1), enriched in synaptic genes and *positively* associated with NFT. *TAGLN3* is another gene with strong NFT association and a highly connected member of these neuronal modules, for which there is evidence from a Drosophila model as being a modifier of tau-induced neurodegeneration [9]. *MAPT per se* and a paralog thereof, *MAP4*, are also members of these neuronal co-expression modules; and their brain transcript levels are also *positively* associated with NFT. Another gene with strong NFT association is *PTPN1*, which was shown to increase in the rat hippocampus with aging in parallel with worse cognition and increased tau phosphorylation [32]. Further, PTPN1 inhibition suppressed amyloid ß-induced tau phosphorylation and improved cognition in a mouse model [29]. In our study, *PTPN1* is negatively associated with NFT, which may reflect a negative feedback from higher protein levels in PSP brains with higher NFT burden, although this remains to be established. These collective experimental findings, which implicate some of the most strongly NFT-associated genes from our study in aspects of tau biology, provide external validation for our findings.

The positive association of *higher* levels of synaptic genes in the temporal cortex with *higher* overall burden of NFT pathology could have several implications. It is possible that regulatory genetic variants result in *higher* levels of synaptic genes, some of which subsequently lead to *higher* neuronal tau accumulation and ultimately risk for PSP. The significant enrichment of the synaptic module in this study for candidate PSP genes proximate to the PSP GWAS [26] risk variants (*MAPT, NSF, CRHR1, STX6, IER5, BMS1*) is in support of this hypothesis. This is also consistent with our prior findings which showed association of PSP risk variants, including *MAPT* locus SNP rs242557, with higher levels of PSP neuropathology [2]. Another explanation for our findings would be that synaptic transcripts are upregulated *in response* to NFT pathology or its consequences, including neuron and synaptic protein loss. Since we measured brain transcripts in the temporal cortex, which is relatively spared from PSP neuropathology, a direct local influence of NFT pathology on expression levels is unlikely in this study. Nevertheless, extracellular release of tau from affected brain regions and its propagation [36] to other brain regions, which may in turn lead to downstream regulatory events, remains theoretically possible. The consistent direction of transcript associations for NFT and TAUTh supports the notion that the same mechanism may underlie the expression associations for these neuronal and extrasomal tau pathologies, respectively. Notably, tau-mediated modification of chromatin structure resulting in aberrant increases in gene expression has been demonstrated in a Drosophila model [20], providing a direct potential link between tau and transcriptional dysregulation. Whether the same epigenetic mechanism underlies the expression changes observed in our study requires further investigation in cohorts of PSP and other primary tauopathies. Our findings demonstrate a significant enrichment for tau neuropathology-associated transcripts which reside on certain chromosomes [Fig. 5, suppl. Table 16 (Online Resource 1)]. This finding, reminiscent of the concept of topologically associated domains [28], may implicate a role for chromatin re-organization in PSP as a potential mechanism for the observed transcriptional changes.

A key finding in our study is the divergent patterns of expression associations especially between the neuronal NFT and astrocytic TA neuropathologies [Figs. 1, 2, suppl. Fig. 4 (Online Resource 2)]. In stark contrast to the NFT associations, *higher* TA pathology is associated with *lower* synaptic gene levels. TA is also the only neuropathology which is not associated with the synaptic co-expression network. Furthermore, levels of immune system transcripts and the immune network (CohortA_M6) are *positively* associated with TA. In contrast, NFT levels are *negatively* associated with immune system transcripts and lack association with the immune network. These findings suggest that different pathomechanisms may underlie the cell-specific tau pathology observed in neurons vs. astrocytes. Indeed, this is consistent with the mismatch observed in PSP between regions of most severe neuronal pathology (e.g., deep nuclei including subthalamic nucleus and substantia nigra) vs. those with most severe astrocytic pathology (e.g., motor cortex and caudate nucleus) [36], which provides neuropathologic evidence for cell-specific vulnerability.

Our findings suggest that aberrant immune transcript expression may specifically underlie the astrocytic tau pathology in PSP [Fig. 3, suppl. Tables 15, 18 (Online Resource 1)]. The immune module, CohortA_M6, which is positively associated with TA pathology is highly enriched in microglial genes. Although there is activation of both microglia and astrocytes following injury to the central nervous system [21], TA pathology was shown to be independent of and therefore likely a different phenomenon than reactive astrogliosis [55]. Microglia were shown to lead to tau aggregation and propagation in neurons in animal models [6, 38], however, their direct influence on astrocytic tau pathology has not been demonstrated. Our findings provide evidence for a link between aberrant upregulation of microglial transcripts and increased astrocytic tau pathology in PSP. Another explanation for these findings may be that upregulation of microglial transcriptional networks may confer relative protection against development of NFT. Increased brain levels of the microglial gene *TREM2* having a protective effect is an emerging concept in AD [11, 35, 59]. Whether this phenomenon is applicable to other microglial network genes and neurodegenerative diseases, such as PSP, remains to be investigated.

In this study, we identified transcriptional associations enriched for other biological processes including the olfactory network (CohortA_M3), which is *negatively* associated with NFT and TAUTh pathologies (Figs. 3, 4, Table 2). Although olfactory dysfunction is not as prominent a clinical feature in PSP compared to other neurodegenerative diseases, including idiopathic Parkinson’s disease, early studies still showed a decline in odor detection in PSP compared to controls [18]. Brain regions particularly associated with olfactory function, such as forebrain cholinergic neurons are shown to harbor NFT pathology but typically exhibit less cell loss in PSP [36], which correlates with the lesser olfactory dysfunction in this condition. Nevertheless, our findings suggest a direct *negative* influence of tau neuropathology on CNS cells involved in odor detection, which is plausible considering the vast complexity of the neurotransmitter networks involved in olfaction [18]. This finding has clinical implications including interrogating olfaction-related neurotransmitter systems in clinical PSP using functional imaging and investigating the utility of odor tests in this condition as potential biomarkers.

It is important to emphasize that the transcriptional and neuropathology measurements evaluated in these PSP brain cohorts represent a single and terminal snapshot in a complex, chronic condition. The transcriptional changes and their associated cell-specific pathologies may therefore reflect differential vulnerability of the specific cells at various stages of the disease. The fact that we are able to discern in this study these divergent patterns of cell-specific neuropathology and brain transcriptional associations may be due to the heterogeneity of the autopsied cohort with respect to disease stage. Another possibility is the presence of different stages of neuropathology in different regions of the same brain samples, with more vulnerable regions harboring a later stage pathology; and less vulnerable regions displaying earlier stage lesions. Our findings support a model where microglial transcriptional upregulation is an earlier event that preferentially influences astrocytic tau pathology, whereas upregulation of synaptic transcripts and concomitant downregulation of vulnerable neuronal pathway genes, such as those involved in olfaction, is a later process in the disease. This model is consistent with the mismatch of lesser neuronal loss in areas most affected with TA; and higher neuronal loss correlating with NFTs. There are other equally compelling models that can fit our results, which require downstream validations in model systems.

This is the first transcriptome-wide association study with tau lesions in PSP, and nominates novel genes and pathways that underlie distinct aspects of tau neuropathology. Our findings suggest that different mechanisms may drive cell-specific tau pathology in a divergent fashion in neurons vs. astrocytes. These results provide a platform for further studies into the divergent biology of different tau neuropathologies in PSP and should serve as a valuable resource for the research community.

## Electronic supplementary material

Below is the link to the electronic supplementary material.

Supplementary material 1 (XLSX 138375 kb)

Supplementary material 2 (DOCX 16920 kb)

Supplementary material 3 (DOCX 169 kb)

## References

[1] The Gene Ontology Consortium (2015). Gene Ontology Consortium: going forward. Nucleic Acids Res.

[2] Allen M, Burgess JD, Ballard T, Serie D, Wang X, Younkin CS, Sun Z, Kouri N, Baheti S, Wang C (2016). Gene expression, methylation and neuropathology correlations at progressive supranuclear palsy risk loci. Acta Neuropathol.

[3] Allen M, Carrasquillo MM, Funk C, Heavner BD, Zou F, Younkin CS, Burgess JD, Chai HS, Crook J, Eddy JA (2016). Human whole genome genotype and transcriptome data for Alzheimer’s and other neurodegenerative diseases. Sci Data.

[4] Allen M, Kachadoorian M, Quicksall Z, Zou F, Chai HS, Younkin C, Crook JE, Pankratz VS, Carrasquillo MM, Krishnan S (2014). Association of MAPT haplotypes with Alzheimer’s disease risk and MAPT brain gene expression levels. Alzheimers Res Ther.

[5] Allen M, Wang X, Burgess JD, Watzlawik J, Serie DJ, Younkin CS, Nguyen T, Malphrus KG, Lincoln S, Carrasquillo MM (2017). Conserved brain myelination networks are altered in Alzheimer’s and other neurodegenerative diseases. Alzheimers Dement.

[6] Asai H, Ikezu S, Tsunoda S, Medalla M, Luebke J, Haydar T, Wolozin B, Butovs.ky O, Kugler S, Ikezu T (2015). Depletion of microglia and inhibition of exosome synthesis halt tau propagation. Nat Neurosci.

[7] Ashburner M, Ball CA, Blake JA, Botstein D, Butler H, Cherry JM, Davis AP, Dolinski K, Dwight SS, Eppig JT (2000). Gene ontology: tool for the unification of biology. The gene ontology consortium. Nat Genet.

[8] Bekpen C, Tastekin I, Siswara P, Akdis CA, Eichler EE (2012). Primate segmental duplication creates novel promoters for the LRRC37 gene family within the 17q21.31 inversion polymorphism region. Genome Res.

[9] Blard O, Feuillette S, Bou J, Chaumette B, Frebourg T, Campion D, Lecourtois M (2007). Cytoskeleton proteins are modulators of mutant tau-induced neurodegeneration in Drosophila. Hum Mol Genet.

[10] Bower JH, Maraganore DM, McDonnell SK, Rocca WA (1997). Incidence of progressive supranuclear palsy and multiple system atrophy in Olmsted County, Minnesota, 1976–1990. Neurology.

[11] Carrasquillo MM, Allen M, Burgess JD, Wang X, Strickland SL, Aryal S, Siuda J, Kachadoorian ML, Medway C, Younkin CS (2016). A candidate regulatory variant at the TREM gene cluster associates with decreased Alzheimer’s disease risk and increased TREML1 and TREM2 brain gene expression. Alzheimers Dement.

[12] Carrasquillo MM, Zou F, Pankratz VS, Wilcox SL, Ma L, Walker LP, Younkin SG, Younkin CS, Younkin LH, Bisceglio GD (2009). Genetic variation in PCDH11X is associated with susceptibility to late-onset Alzheimer’s disease. Nat Genet.

[13] Cox J, Neuhauser N, Michalski A, Scheltema RA, Olsen JV, Mann M (2011). Andromeda: a peptide search engine integrated into the maxquant environment. J Proteome Res.

[14] Coyle-Gilchrist IT, Dick KM, Patterson K, Vazquez Rodriquez P, Wehmann E, Wilcox A, Lansdall CJ, Dawson KE, Wiggins J, Mead S (2016). Prevalence, characteristics, and survival of frontotemporal lobar degeneration syndromes. Neurology.

[15] Cruz JC, Tseng HC, Goldman JA, Shih H, Tsai LH (2003). Aberrant Cdk5 activation by p25 triggers pathological events leading to neurodegeneration and neurofibrillary tangles. Neuron.

[16] Das S, Forer L, Schonherr S, Sidore C, Locke AE, Kwong A, Vrieze SI, Chew EY, Levy S, McGue M (2016). Next-generation genotype imputation service and methods. Nat Genet.

[17] Dickson DW, Rademakers R, Hutton ML (2007). Progressive supranuclear palsy: pathology and genetics. Brain Pathol.

[18] Doty RL (2017). Olfactory dysfunction in neurodegenerative diseases: is there a common pathological substrate?. Lancet Neurol.

[19] Du P, Kibbe WA, Lin SM (2008). lumi: a pipeline for processing Illumina microarray. Bioinformatics.

[20] Frost B, Hemberg M, Lewis J, Feany MB (2014). Tau promotes neurodegeneration through global chromatin relaxation. Nat Neurosci.

[21] Gao Z, Zhu Q, Zhang Y, Zhao Y, Cai L, Shields CB, Cai J (2013). Reciprocal modulation between microglia and astrocyte in reactive gliosis following the CNS injury. Mol Neurobiol.

[22] Gerschutz A, Heinsen H, Grunblatt E, Wagner AK, Bartl J, Meissner C, Fallgatter AJ, Al-Sarraj S, Troakes C, Ferrer I (2014). Neuron-specific alterations in signal transduction pathways associated with Alzheimer’s disease. J Alzheimers Dis.

[23] Grubert F, Zaugg JB, Kasowski M, Ursu O, Spacek DV, Martin AR, Greenside P, Srivas R, Phanstiel DH, Pekowska A (2015). Genetic Control of chromatin states in humans involves local and distal chromosomal interactions. Cell.

[24] Hauw JJ, Daniel SE, Dickson D, Horoupian DS, Jellinger K, Lantos PL, McKee A, Tabaton M, Litvan I (1994). Preliminary NINDS neuropathologic criteria for Steele–Richardson–Olszewski syndrome (progressive supranuclear palsy). Neurology.

[25] Heimer G, Marek-Yagel D, Eyal E, Barel O, Oz Levi D, Hoffmann C, Ruzzo EK, Ganelin-Cohen E, Lancet D, Pras E (2015). SLC1A4 mutations cause a novel disorder of intellectual disability, progressive microcephaly, spasticity and thin corpus callosum. Clin Genet.

[26] Hoglinger GU, Melhem NM, Dickson DW, Sleiman PM, Wang LS, Klei L, Rademakers R, de Silva R, Litvan I, Riley DE (2011). Identification of common variants influencing risk of the tauopathy progressive supranuclear palsy. Nat Genet.

[27] Hong Y, Chan CB, Kwon IS, Li X, Song M, Lee HP, Liu X, Sompol P, Jin P, Lee HG (2012). SRPK2 phosphorylates tau and mediates the cognitive defects in Alzheimer’s disease. J Neurosci.

[28] Hu Z, Tee WW (2017). Enhancers and chromatin structures: regulatory hubs in gene expression and diseases. Biosci Rep.

[29] Kanno T, Tsuchiya A, Tanaka A, Nishizaki T (2016). Combination of PKCepsilon activation and PTP1B inhibition effectively suppresses abeta-induced GSK-3beta activation and tau phosphorylation. Mol Neurobiol.

[30] Koga S, Dickson DW, Bieniek KF (2016). Chronic traumatic encephalopathy pathology in multiple system atrophy. J Neuropathol Exp Neurol.

[31] Kouri N, Ross OA, Dombroski B, Younkin CS, Serie DJ, Soto-Ortolaza A, Baker M, Finch NC, Yoon H, Kim J (2015). Genome-wide association study of corticobasal degeneration identifies risk variants shared with progressive supranuclear palsy. Nat Commun.

[32] Kuga GK, Munoz VR, Gaspar RC, Nakandakari S, da Silva ASR, Botezelli JD, Leme J, Gomes RJ, de Moura LP, Cintra DE (2018). Impaired insulin signaling and spatial learning in middle-aged rats: the role of PTP1B. Exp Gerontol.

[33] Langfelder P, Horvath S (2012). Fast R functions for robust correlations and hierarchical clustering. J Stat Softw.

[34] Langfelder P, Horvath S (2008). WGCNA: an R package for weighted correlation network analysis. BMC Bioinformatics.

[35] Lee CYD, Daggett A, Gu X, Jiang LL, Langfelder P, Li X, Wang N, Zhao Y, Park CS, Cooper Y (2018). Elevated TREM2 gene dosage reprograms microglia responsivity and ameliorates pathological phenotypes in Alzheimer’s disease models. Neuron.

[36] Lewis J, Dickson DW (2016). Propagation of tau pathology: hypotheses, discoveries, and yet unresolved questions from experimental and human brain studies. Acta Neuropathol.

[37] Luciano M, Hansell NK, Lahti J, Davies G, Medland SE, Raikkonen K, Tenesa A, Widen E, McGhee KA, Palotie A (2011). Whole genome association scan for genetic polymorphisms influencing information processing speed. Biol Psychol.

[38] Maphis N, Xu G, Kokiko-Cochran ON, Jiang S, Cardona A, Ransohoff RM, Lamb BT, Bhaskar K (2015). Reactive microglia drive tau pathology and contribute to the spreading of pathological tau in the brain. Brain.

[39] Murray ME, Kouri N, Lin WL, Jack CR, Dickson DW, Vemuri P (2014). Clinicopathologic assessment and imaging of tauopathies in neurodegenerative dementias. Alzheimers Res Ther.

[40] Patrick GN, Zukerberg L, Nikolic M, de la Monte S, Dikkes P, Tsai LH (1999). Conversion of p35 to p25 deregulates Cdk5 activity and promotes neurodegeneration. Nature.

[41] Pearl JR, Bergey DE, Funk CC, Basu B, Oshone R, Shannon P, Hood L, Price ND, Colantuoni C, Ament SA (2017). Genome-scale transcriptional regulatory network models of psychiatric and neurodegenerative disorders. bioRxiv.

[42] Pittman AM, Fung HC, de Silva R (2006). Untangling the tau gene association with neurodegenerative disorders. Hum Mol Genet.

[43] Purcell S, Neale B, Todd-Brown K, Thomas L, Ferreira MA, Bender D, Maller J, Sklar P, de Bakker PI, Daly MJ (2007). PLINK: a tool set for whole-genome association and population-based linkage analyses. Am J Hum Genet.

[44] Rizo J, Xu J (2015). The synaptic vesicle release machinery. Annu Rev Biophys.

[45] Rizopoulos D (2006). ltm: an R Package for latent variable modeling and item response theory analyses. J Stat Softw.

[46] Schlicker A, Domingues FS, Rahnenfuhrer J, Lengauer T (2006). A new measure for functional similarity of gene products based on gene ontology. BMC Bioinformatics.

[47] Simon-Sanchez J, Schulte C, Bras JM, Sharma M, Gibbs JR, Berg D, Paisan-Ruiz C, Lichtner P, Scholz SW, Hernandez DG (2009). Genome-wide association study reveals genetic risk underlying Parkinson’s disease. Nat Genet.

[48] Srinivasan K, Friedman BA, Larson JL, Lauffer BE, Goldstein LD, Appling LL, Borneo J, Poon C, Ho T, Cai F (2016). Untangling the brain’s neuroinflammatory and neurodegenerative transcriptional responses. Nat Commun.

[49] Srour M, Hamdan FF, Gan-Or Z, Labuda D, Nassif C, Oskoui M, Gana-Weisz M, Orr-Urtreger A, Rouleau GA, Michaud JL (2015). A homozygous mutation in SLC1A4 in siblings with severe intellectual disability and microcephaly. Clin Genet.

[50] Steinberg KM, Antonacci F, Sudmant PH, Kidd JM, Campbell CD, Vives L, Malig M, Scheinfeldt L, Beggs W, Ibrahim M (2012). Structural diversity and African origin of the 17q21.31 inversion polymorphism. Nat Genet.

[51] Storey JD, Tibshirani R (2003). Statistical significance for genome-wide experiments. Proc Natl Acad Sci.

[52] Sundermann F, Fernandez MP, Morgan RO (2016). An evolutionary roadmap to the microtubule-associated protein MAP tau. BMC Genomics.

[53] Supek F, Bosnjak M, Skunca N, Smuc T (2011). REVIGO summarizes and visualizes long lists of gene ontology terms. PLoS One.

[54] Takahashi M, Weidenheim KM, Dickson DW, Ksiezak-Reding H (2002). Morphological and biochemical correlations of abnormal tau filaments in progressive supranuclear palsy. J Neuropathol Exp Neurol.

[55] Togo T, Dickson DW (2002). Tau accumulation in astrocytes in progressive supranuclear palsy is a degenerative rather than a reactive process. Acta Neuropathol.

[56] Wickham H (2009). Ggplot2: elegant graphics for data analysis.

[57] Willer CJ, Li Y, Abecasis GR (2010). METAL: fast and efficient meta-analysis of genomewide association scans. Bioinformatics.

[58] Zhao Y, Tseng IC, Heyser CJ, Rockenstein E, Mante M, Adame A, Zheng Q, Huang T, Wang X, Arslan PE (2015). Appoptosin-mediated caspase cleavage of tau contributes to progressive supranuclear palsy pathogenesis. Neuron.

[59] Zhao Y, Wu X, Li X, Jiang LL, Gui X, Liu Y, Sun Y, Zhu B, Pina-Crespo JC, Zhang M (2018). TREM2 is a receptor for beta-amyloid that mediates microglial function. Neuron.

[60] Zou F, Chai HS, Younkin CS, Allen M, Crook J, Pankratz VS, Carrasquillo MM, Rowley CN, Nair AA, Middha S (2012). Brain expression genome-wide association study (eGWAS) identifies human disease-associated variants. PLoS Genet.

