# Supplementary material for: Divergent brain gene expression patterns associate with distinct cell-specific tau neuropathology traits in progressive supranuclear palsy
Source: Acta Neuropathol. 2018 Aug 22;136(5):709–27. doi: 10.1007/s00401-018-1900-5 (PMC6208732; doi:10.1007/s00401-018-1900-5)
Supplement: Supplementary file 2 — Supplementary material 2 (DOCX 16920 kb) [file 401_2018_1900_MOESM2_ESM.docx]

**Electronic Supplementary Material**

**Divergent brain gene expression patterns associate with distinct cell-specific tau neuropathology traits in progressive supranuclear palsy**

**Authors**

Mariet Allen^1^, Xue Wang^2^, Daniel J. Serie^2^, Samantha L. Strickland^1^, Jeremy D. Burgess^1^, Shunsuke Koga^1^, Curtis S. Younkin^3^, Thuy T. Nguyen^1^, Kimberly G. Malphrus^1^, Sarah J. Lincoln^1^, Melissa Alamprese^4^, Kuixi Zhu^5^, Rui Chang^5,6^, Minerva M. Carrasquillo^1^, Naomi Kouri^1^, Melissa E. Murray^1^, Joseph S. Reddy^2^, Cory Funk^7^, Nathan D. Price^7^, Todd E. Golde^8^, Steven G. Younkin^1^, Yan W. Asmann^2^, Julia E. Crook^2^, Dennis W. Dickson^1^, Nilüfer Ertekin-Taner^1,9, #^

**Author Affiliations:**

1) Mayo Clinic, Department of Neuroscience, Jacksonville, FL 32224 USA

2) Mayo Clinic, Department of Health Sciences Research, Jacksonville, FL 32224 USA

3) Mayo Clinic, Division of Information Technology, Jacksonville, FL 32224 USA.

4) Banner Behavior Health, Phoenix AZ 85016 USA

5) University of Arizona, The Center for Innovation in Brain Sciences, Tucson AZ 85721 USA

6) University of Arizona, Department of Neurology, Tucson AZ 85721 USA

7) Institute for Systems Biology, 401 Terry Avenue N, Seattle, WA 98109, USA.

8) Center for Translational Research in Neurodegenerative Disease, McKnight Brain Institute, University of Florida, Department of Neuroscience, Gainesville, FL 32610, USA.

9) Mayo Clinic, Department of Neurology, Jacksonville, FL 32224 USA.

# Corresponding Author

**Corresponding Author Contact Information:** Mayo Clinic, Departments of Neurology and Neuroscience, 4500 San Pablo Road, Birdsall 3, Jacksonville, FL 32224.

E-mail: taner.nilufer@mayo.edu, Phone: 904-953-7103, FAX: 904-953-7353.

**Contents:**

Supplementary Figures 1-12


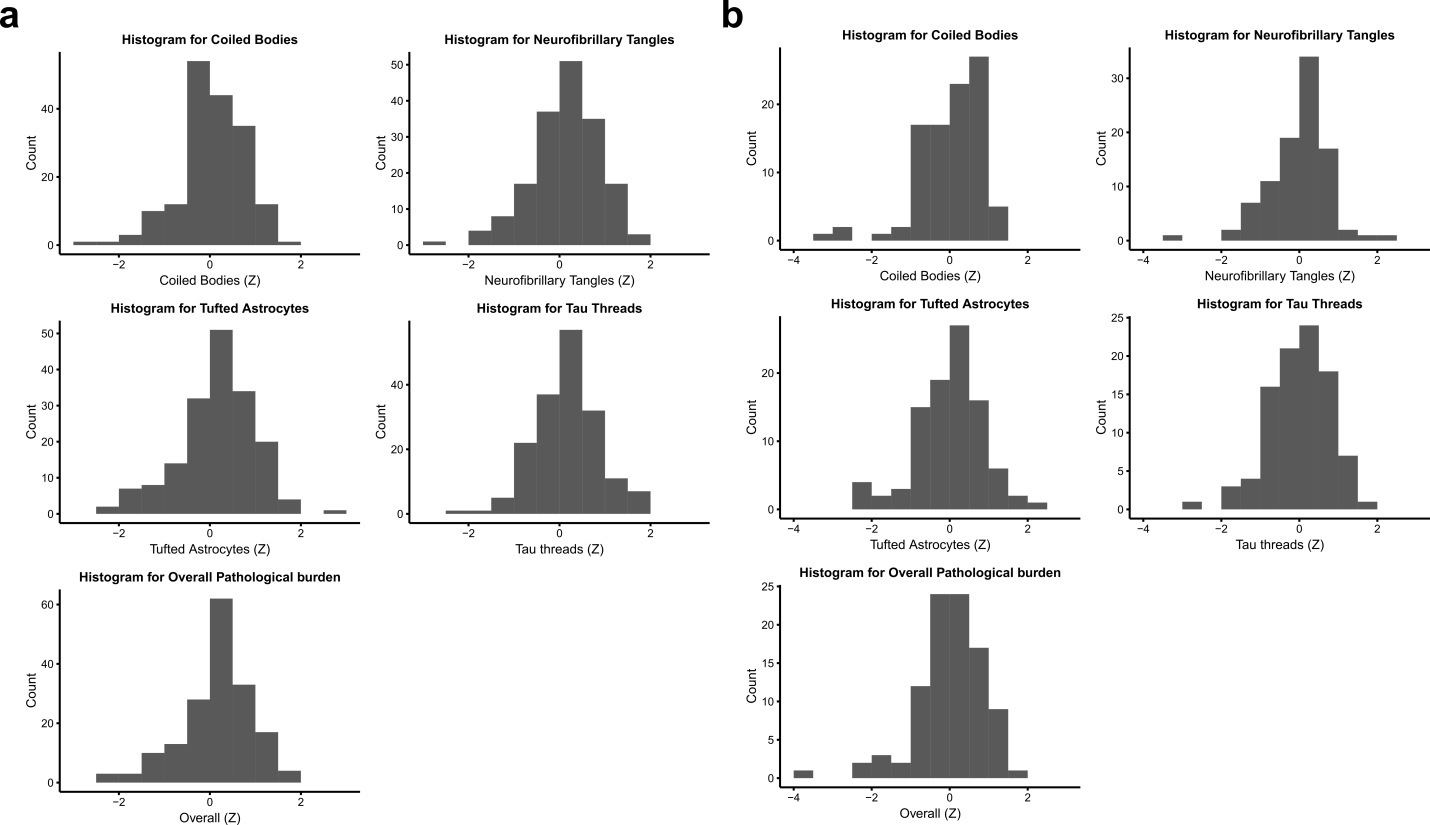


**suppl. figure 1**. **Histograms of neuropathology latent traits:** Histograms of the continuous neuropathology latent trait residuals adjusted for age and sex are shown. Distribution of each neuropathological trait is shown separately for the two cohorts: **a** = Cohort A, **b** = Cohort B. The x axis indicates the residual value and the y axis indicates the number of samples within the range of values represented by the bar.


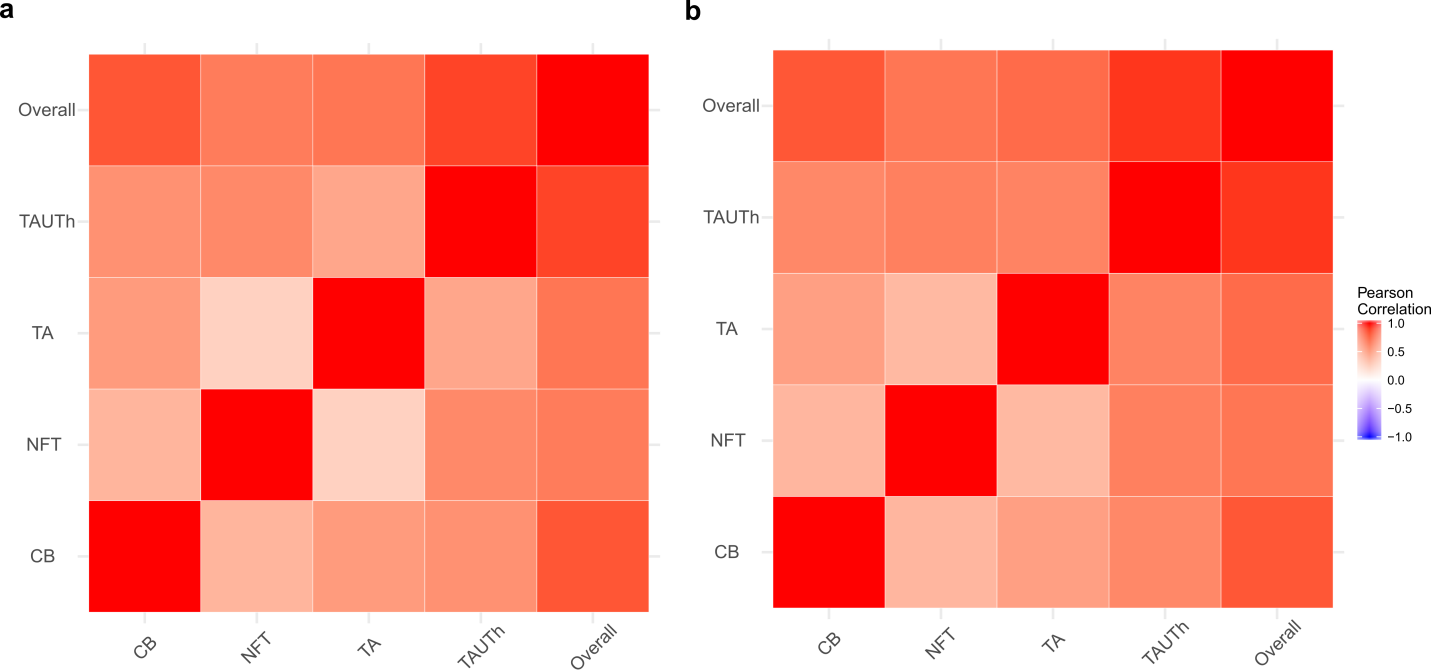

**suppl. figure 2**. **Latent and regional semi-quantitative neuropathology traits:** (**a**) and (**b**) Heat maps of Pearson correlation coefficients for each pairwise latent neuropathology trait**.** Correlation coefficients are plotted for the neuropathology trait residuals separately for Cohort A (**a**) and Cohort B (**b**). (**c**) Regional semi-quantitative neuropathology trait averages for Cohort A are shown for each of the 19 regions, which are used to estimate the latent traits, as well as the temporal cortex, which is typically less affected by PSP neuropathology and used in the transcriptome measurements. Regional semi-quantitative neuropathology count averages are highlighted from from lighter to darker hues to reflect average neuropathology counts of 1-2, 2-3 and >3, respectively. CB = Coiled Bodies, NFT = Neurofibrillary Tangles, TA = Tufted Astrocytes, TAUTh = Tau threads.


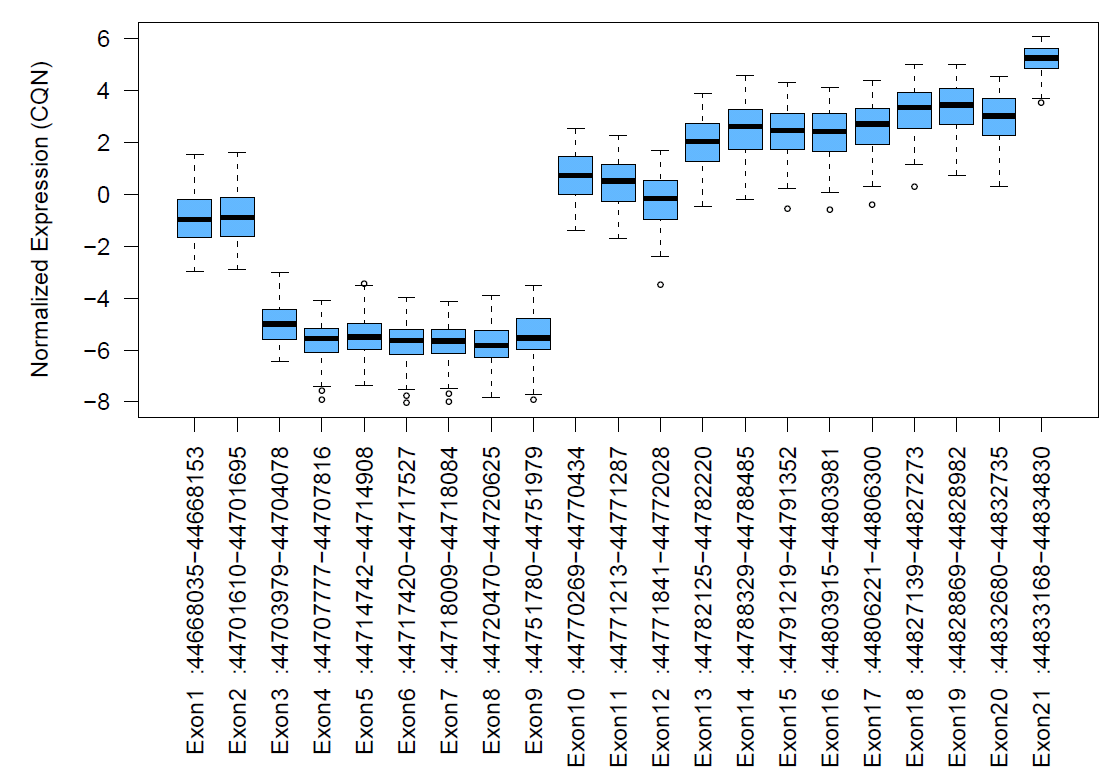


**suppl. figure 3**. **Expression levels of NSF exons:** Using “Mayo Clinic RNAseq study” PSP data (n=82) described in[[1](#_ENREF_1)] and available at AMP-AD knowledge portal (doi:10.7303/syn2580853; syn6090813), we obtained expression levels of NSF exons, defined according to transcript NM_006178 (ENSEMBL ID: ENST00000398238.4). Conditional Quantile Normalized (CQN) read counts for each exon are plotted as box plots. Line within box=median, top and bottom of box=1^st^ and 3^rd^ quartiles, respectively, whiskers=1.5x inter-quartile range, circles=outliers.


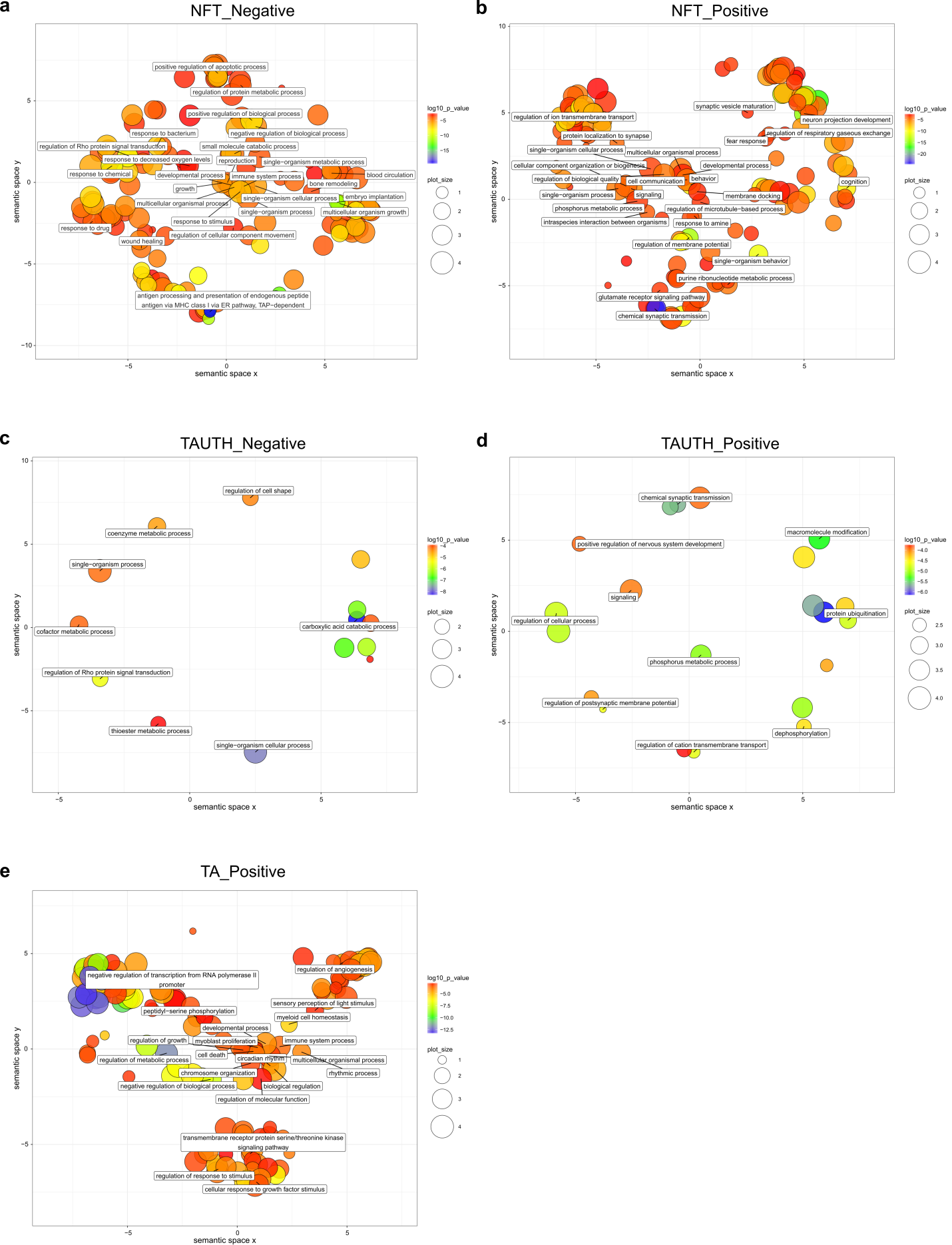


**suppl. figure 4. REVIGO scatter plots of significant GO terms for genes with expression-neuropathology associations:** REVIGO plots clustering significant (FDR<0.05) GO terms in 2-dimensional space according to semantic similarities are generated for associated genes with >20 significant GO terms. Bubble color reflects the enrichment p-value; the size of the bubble refers to the size of the GO term; labeled bubbles are the most unique terms in the cluster. Plots were generated for GO terms enriched in genes negatively associated with NFT (**a**), positively associated with NFT (**b**), negatively associated with TAUTh (**c**), positively associated with TAUTh (**d**) and positively associated with TA (**e**).

**suppl. figure 5. Topological overlap matrix (TOM) in Cohort A:** Heatmap of pairwise correlations for 2000 randomly selected probes are shown to visualize within and between module clustering.


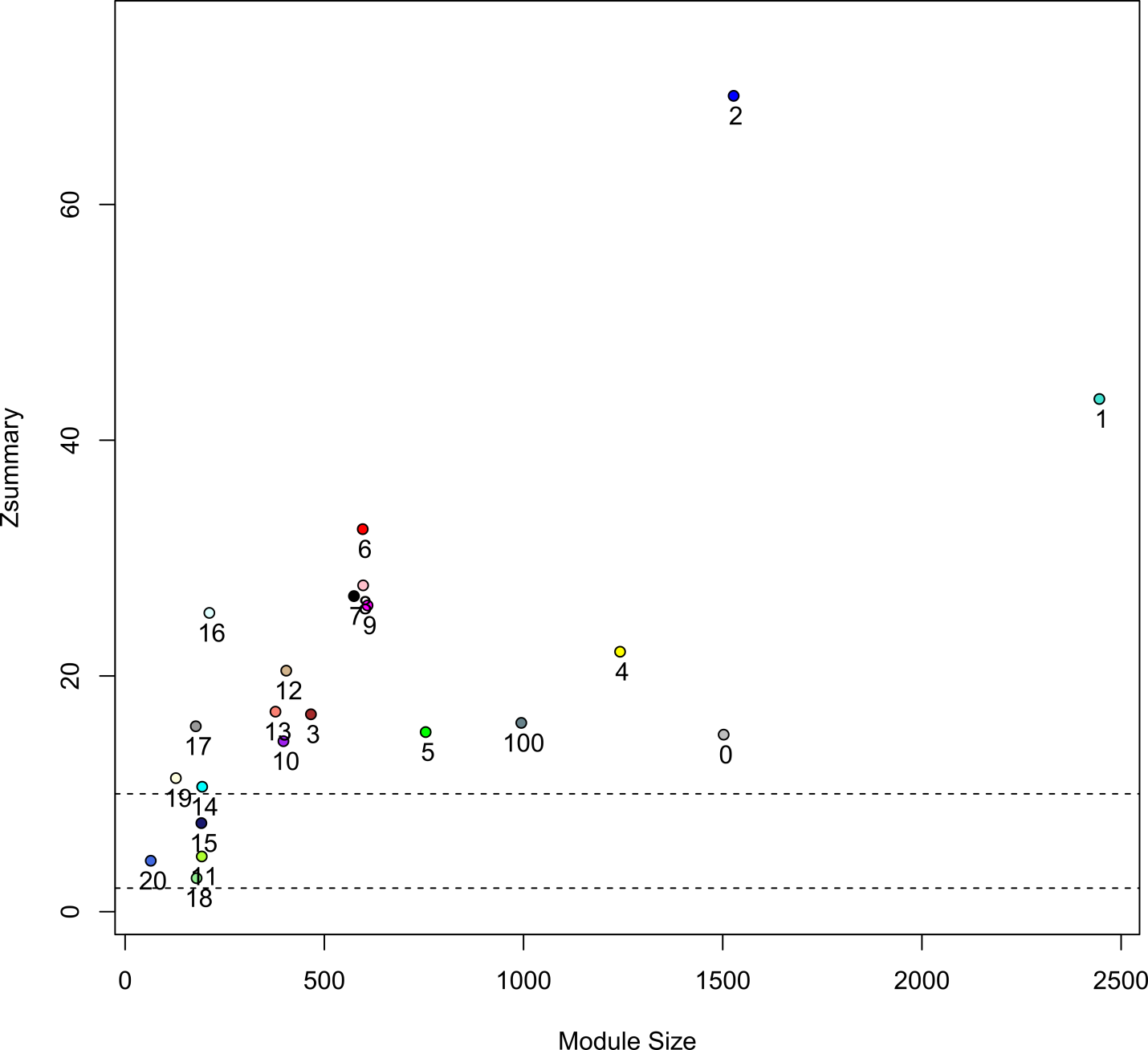


**suppl. figure 6. Preservation of co-expression modules from Cohort A in Cohort B:** WGCNA “modulePreservation” function with 100 permutations was applied to calculate preservation statistic Zsummary for a specific module’s genes in comparison groups. Zsummary score (y-axis) vs. CEM size (x-Axis) are plotted. Typically, a Zsummary score >10 indicates that the module genes were well-preserved. Modules CohortA_M2, CohortA_M3 and CohortA_M13 are all well preserved in Cohort B.


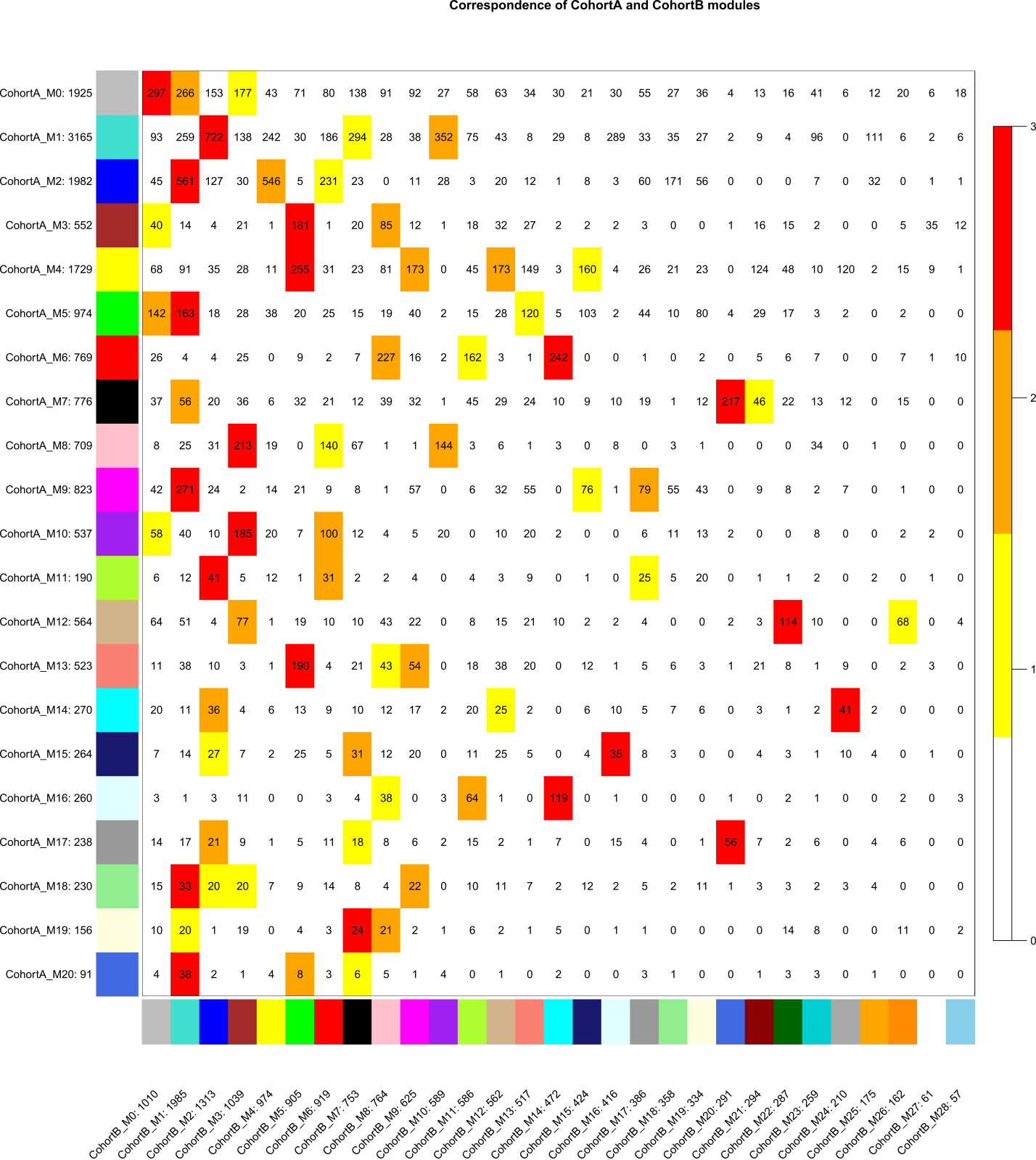


**suppl. figure 7. Overlap of transcripts in the co-expression modules from Cohort A and Cohort B:** Matrix for the number of overlapping transcripts in co-expression modules from Cohort A (y-axis) and Cohort B (x-axis). Coloring palette to the right depicts the modules with the first, second and third highest number of overlapping transcripts.


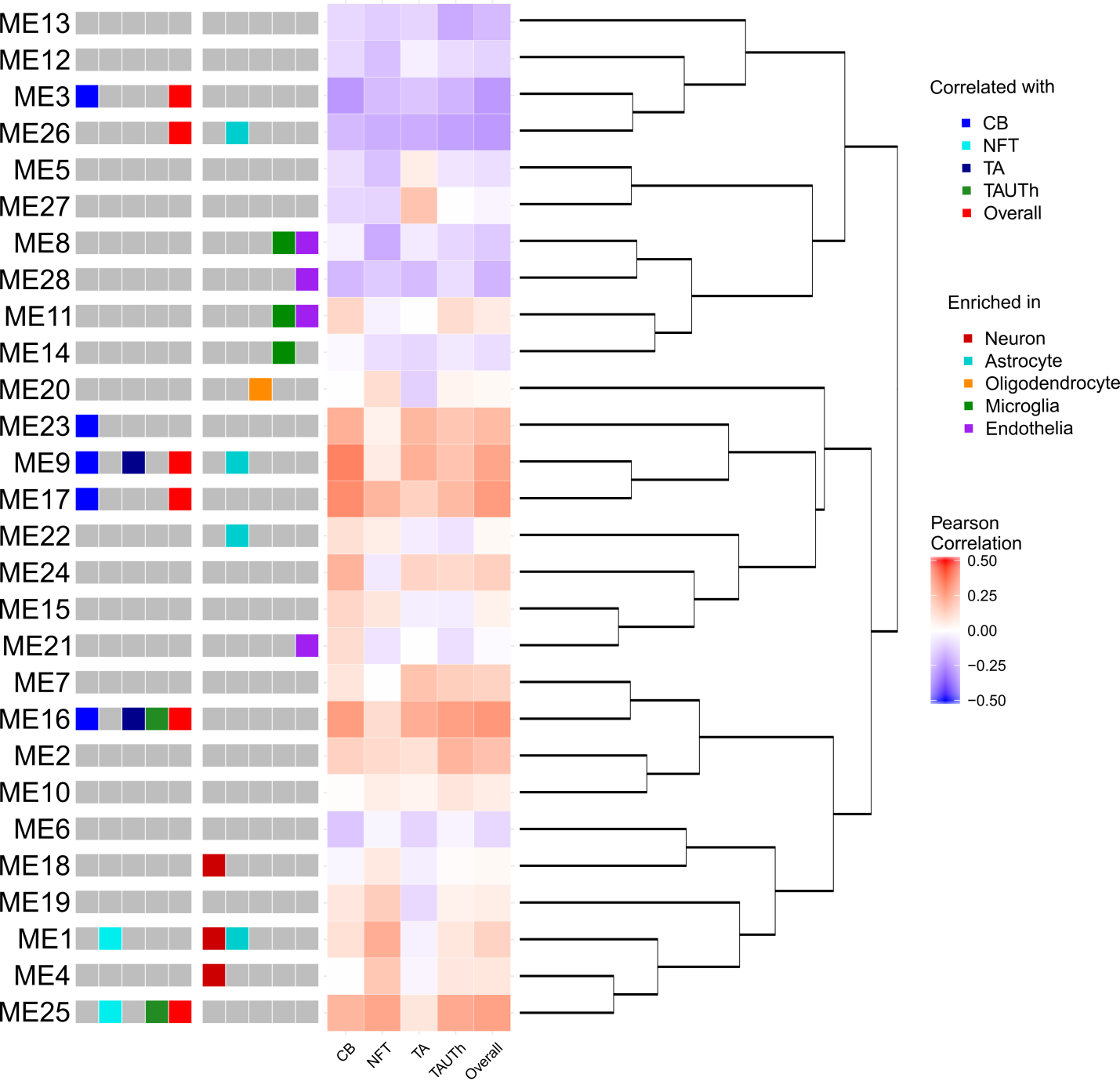


**suppl. figure 8. Cohort B co-expression modules:** Neuropathology association, CNS cell type enrichment and module hierarchical clustering results are depicted for the co-expression modules from Cohort B. Left to right: Neuropathology barcode indicating module eigengenes (MEs) nominally associated (unadjusted p< 0.05) with CB, NFT, TA, TAUTh or Overall traits according to the legend in the top right; Cell type enrichment barcode indicating modules enriched for genes that are predominantly expressed in CNS cell types, color coded according to the middle right legend; Heatmap illustrating the Pearson correlation for each ME with the neuropathological traits, Bonferroni significant ME’s are indicating with a star (no Bonferroni-significant module-neuropathology associations were detected for Cohort B, after correcting for 28 tests); hierarchical clustering dendrogram depicting the relationship between MEs.


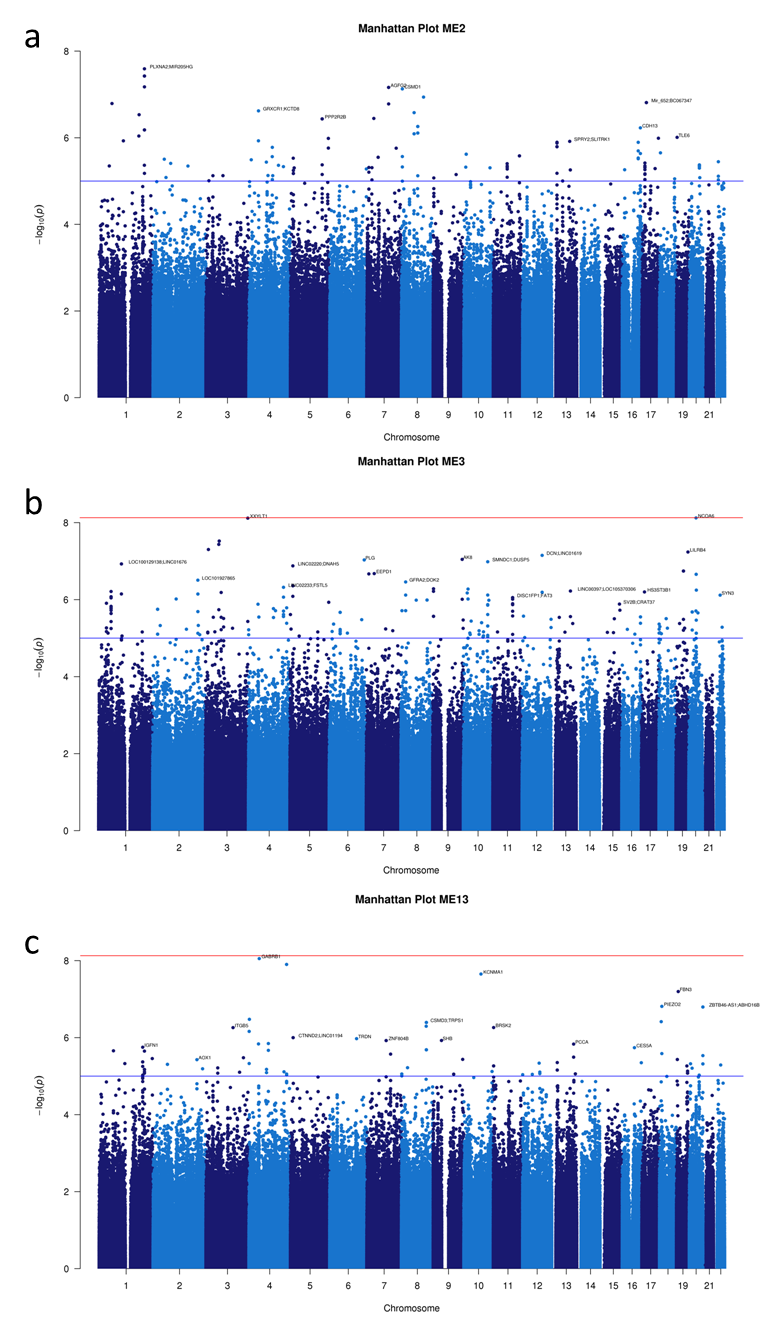


**suppl. figure 9. Manhattan plots for module QTL associations:** Results are shown for genome-wide variants with module eigengenes of CohortA_M2 (**a**), CohortA_M3 (**b**) and CohortA_M13 (**c**). The x axis represents chromosome and positon, and the Y axis the log10 p-value.


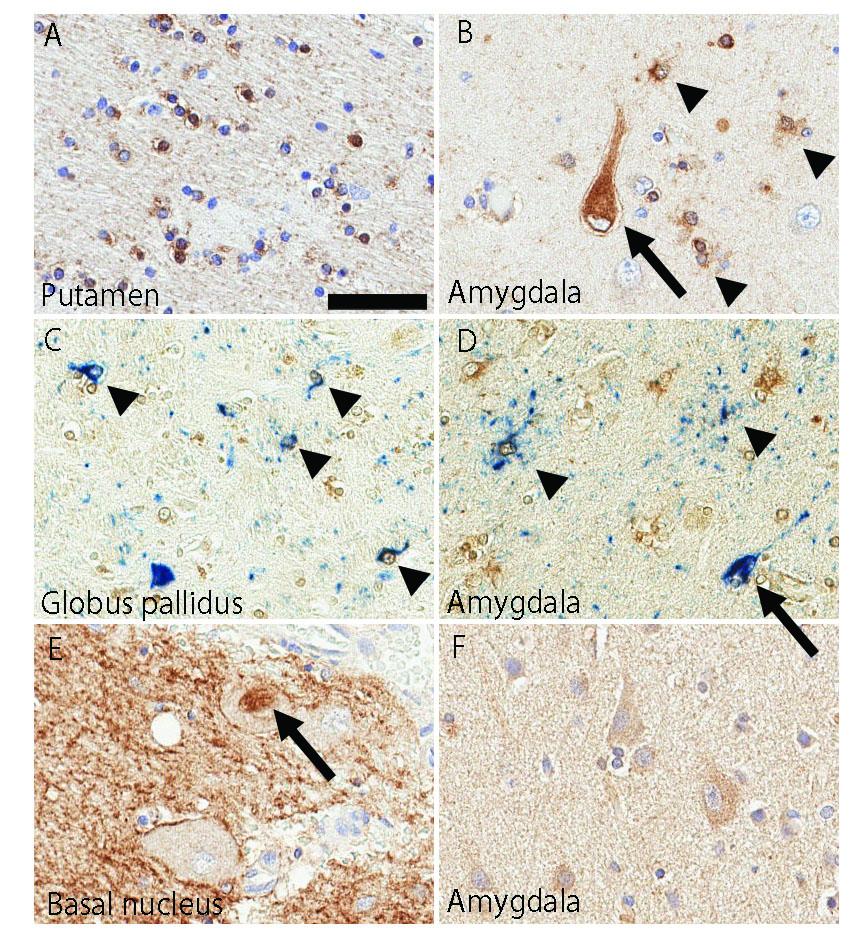


**suppl. figure 10. Representative images of immunohistochemistry** for MAP4, SLC1A4, and NSF in PSP brains. MAP4 is expressed in oligodendrocytes (A), astrocytes (B, arrow heads), and a subset of neurons (B, arrow). Double-labelling immunohistochemistry with MAP4 (brown) and tau (blue) shows that coiled bodies (C, arrow heads), tufted astrocytes (D, arrow heads), and pretangles (D, arrow) express MAP4. SLC1A4 is stained in the neuronal membrane and cell processes, and some neurons in the basal nucleus of Meynert have globose tangle-like staining of SLC1A4 in PSP (E, arrow). NSF is expressed in almost all neuronal cytoplasm in PSP (F). All images are same magnification. Bar = 50 μm.

**References:**

1. Allen M, Carrasquillo MM, Funk C, Heavner BD, Zou F, Younkin CS, Burgess JD, Chai HS, Crook J, Eddy JA, Li H, Logsdon B, Peters MA, Dang KK, Wang X, Serie D, Wang C, Nguyen T, Lincoln S, Malphrus K, Bisceglio G, Li M, Golde TE, Mangravite LM, Asmann Y, Price ND, Petersen RC, Graff-Radford NR, Dickson DW, Younkin SG, Ertekin-Taner N (2016) Human whole genome genotype and transcriptome data for Alzheimer's and other neurodegenerative diseases. Sci Data 3:160089. doi:10.1038/sdata.2016.89
