# Supplementary material for: Divergent brain gene expression patterns associate with distinct cell-specific tau neuropathology traits in progressive supranuclear palsy
Source: Acta Neuropathol. 2018 Aug 22;136(5):709–27. doi: 10.1007/s00401-018-1900-5 (PMC6208732; doi:10.1007/s00401-018-1900-5)
Supplement: Supplementary file 3 — Supplementary material 3 (DOCX 169 kb) [file 401_2018_1900_MOESM3_ESM.docx]

**Electronic Supplementary Material**

**Divergent brain gene expression patterns associate with distinct cell-specific tau neuropathology traits in progressive supranuclear palsy**

**Authors**

Mariet Allen^1^, Xue Wang^2^, Daniel J. Serie^2^, Samantha L. Strickland^1^, Jeremy D. Burgess^1^, Shunsuke Koga^1^, Curtis S. Younkin^3^, Thuy T. Nguyen^1^, Kimberly G. Malphrus^1^, Sarah J. Lincoln^1^, Melissa Alamprese^4^, Kuixi Zhu^5^, Rui Chang^5,6^, Minerva M. Carrasquillo^1^, Naomi Kouri^1^, Melissa E. Murray^1^, Joseph S. Reddy^2^, Cory Funk^7^, Nathan D. Price^7^, Todd E. Golde^8^, Steven G. Younkin^1^, Yan W. Asmann^2^, Julia E. Crook^2^, Dennis W. Dickson^1^, Nilüfer Ertekin-Taner^1,9, #^

**Author Affiliations:**

1) Mayo Clinic, Department of Neuroscience, Jacksonville, FL 32224 USA

2) Mayo Clinic, Department of Health Sciences Research, Jacksonville, FL 32224 USA

3) Mayo Clinic, Division of Information Technology, Jacksonville, FL 32224 USA.

4) Banner Behavior Health, Phoenix AZ 85016 USA

5) University of Arizona, The Center for Innovation in Brain Sciences, Tucson AZ 85721 USA

6) University of Arizona, Department of Neurology, Tucson AZ 85721 USA

7) Institute for Systems Biology, 401 Terry Avenue N, Seattle, WA 98109, USA.

8) Center for Translational Research in Neurodegenerative Disease, McKnight Brain Institute, University of Florida, Department of Neuroscience, Gainesville, FL 32610, USA.

9) Mayo Clinic, Department of Neurology, Jacksonville, FL 32224 USA.

# Corresponding Author

**Corresponding Author Contact Information:** Mayo Clinic, Departments of Neurology and Neuroscience, 4500 San Pablo Road, Birdsall 3, Jacksonville, FL 32224.

E-mail: taner.nilufer@mayo.edu, Phone: 904-953-7103, FAX: 904-953-7353.

**Contents:**

Supplementary Text

**Suppl. Text:**

**Suppl. Methods**

**Microarray expression Cohorts A and B**

Gene expression measures were collected from post-mortem temporal cortex tissue of 268 subjects with a neuropathological diagnosis of PSP (**Methods**). Expression data was collected in two batches: Cohort A comprised of 173 PSP subjects and Cohort B comprised of 95 independent PSP subjects, using Illumina WG-DASL microarrays (**Table 1, Methods)**. Each of these datasets was analyzed separately to avoid batch effects due to differences in content, approach and time of collection.

***Quality Control*:** For Cohort A, expression measures were collected for 192 samples, of which 176 had available genome-wide genotypes that passed quality control[[2](#_ENREF_2)], and were retained for analysis. Three of these samples were removed from the study following expression data QC: Two samples were identified as outliers following principal components analysis and one sample had expression for chromosome Y genes that did not match with the recorded sex; resulting in a final cohort of 173 samples. In Cohort B, 95 samples had a pathological diagnosis of PSP. These samples were part of the Mayo Clinic expression GWAS, for which QC has been described previously[[39](#_ENREF_39)]. All QC of expression data was carried out using R statistical software (R Foundation for Statistical Computing, version 3.2.3) unless otherwise stated. Only those samples that passed both expression data and genetic data QC (see below) were retained in this study.

***Genetic Data*:** Subjects in Cohort A were previously genotyped using Human 660W-Quad Infinium BeadChips (Illumina, San Diego, CA, USA) as part of a published PSP risk genome-wide association study[[15](#_ENREF_15)]. Subjects in Cohort B were previously genotyped using the HumanHap300-Duo Genotyping BeadChips (Illumina, San Diego, CA, USA)[[9](#_ENREF_9)]. For Cohorts A and B, QC based on genome-wide genetic data has been previously described[[2](#_ENREF_2), [9](#_ENREF_9), [39](#_ENREF_39)]. Both cohorts A and B were imputed to the HRC reference panel **(Methods)**.

***Expression quantitative trait (eQTL) analysis***

Probe expression measures for subjects in Cohort A only were assessed for association with imputed genome-wide genotype data[[2](#_ENREF_2), [15](#_ENREF_15)] (“**Genome-wide genotypes”** methods section). Analysis was performed for each probe using multi-variable linear regression (additive model) implemented using PLINK[[27](#_ENREF_27)], including covariates Age, Sex, RIN, PCR plate, RINsqAdj ((RIN-RIN_mean_)^2^) and four principal components previously generated[[2](#_ENREF_2)] using EIGENSOFT[[24](#_ENREF_24), [26](#_ENREF_26)]. Association results for variants that were “*in-cis*”, defined as +/-100kb from the 5’ and 3’ terminal ends of the gene targeted by a given probe were mapped (hg19), extracted and databased, to enable efficient querying.

***Module QTL***

Module eigengenes for Cohort A were assessed for association with imputed genome-wide genotype data[[2](#_ENREF_2), [15](#_ENREF_15)] (“**Genome-wide genotypes”** methods section). Analysis was performed for each eigengene using multi-variable linear regression (additive model) implemented using PLINK[[27](#_ENREF_27)], including four principal components previously generated[[2](#_ENREF_2)] using EIGENSOFT[[24](#_ENREF_24), [26](#_ENREF_26)], as covariates. Association p-values were adjusted for multiple tests using false discovery rate (Benjamini-Hochberg)[[31](#_ENREF_31)]. Manhattan plots were generated using the qqman package (R statistical software).

**Mayo Clinic RNAseq Study**

Gene expression measures were collected using RNA sequencing, for RNA isolated from temporal cortex tissue of 84 PS and 80 controls samples as previously described[[3](#_ENREF_3)]. All PSP subjects in the Mayo Clinic RNAseq study are a subset of those in microarray Cohort A. Control subjects were neuropathologically assessed and had Braak[[8](#_ENREF_8)] NFT stage of 3.0 or less, CERAD[[22](#_ENREF_22)] neuritic and cortical plaque densities of 0 (none) or 1 (sparse) and lacked any of the following pathologic diagnoses: AD, Parkinson’s disease (PD), DLB, VaD, PSP, motor neuron disease (MND), CBD, Pick’s disease (PiD), Huntington’s disease (HD), FTLD, hippocampal sclerosis (HipScl) or dementia lacking distinctive histology (DLDH). Thirty-one control samples were from the Mayo Clinic Brain Bank, and the remaining from the Banner Sun Health Institute**.** Following RNA isolation **(Methods)**, the TruSeq RNA Sample Prep Kit (Illumina, San Diego, CA) was used for library preparation, and library concentration and size distribution was determined on an Agilent Bioanalyzer DNA 1000 chip. Sequencing was performed on the Illumina HiSeq2000 (101 base pair paired-end sequencing) with triplicate multiplexing of barcoded samples per flowcell lane. Base-calling was performed using Illumina’s RTA 1.17.21.3 and FASTQ reads aligned to human genome build 19 using the MAP-RSeq pipeline[[17](#_ENREF_17)], implementing Tophat version 2.0.12 with Bowtie version 1.1.0 and FeatureCounts version 1.4.4. Raw read counts were normalized using Conditional Quantile Normalization (CQN) via the Bioconductor package; accounting for sequencing depth, gene length, and GC content. GC content was calculated via the Bioconductor package, Repitools, and sequencing depth was calculated as the sum of reads mapped to genes.

***Quality Control*:** FastQC was used for quality control of raw sequence reads, and RSeQC was used for QC of mapped reads; where all samples had percent mapped reads ≥ 85%. The mean log2 normalized counts per million (CPM) expression levels of non-zero count chromosome Y genes, were used to identify any samples with deviation from expected expression based on recorded sex. CQN normalized expression measures were assessed using principal components analysis (PCA) to identify outliers defined as greater than 6 standard deviations from the mean of the first two principal components. All PSP and control samples in the Mayo Clinic RNAseq study passed expression data QC. All QC of expression data was carried out using R statistical software (R Foundation for Statistical Computing, version 3.2.3) unless otherwise stated. Only those samples that passed both expression data and genetic data QC (see below) were retained in this study.

***Genetic Data*:** Subjects in the Mayo Clinic RNASeq cohort were genotyped using Omni 2.5 Beadchips (Illumina, San Diego, CA, USA). Samples were assessed, using PLINK[[27](#_ENREF_27)], for discordant sex, low call rate (<98%) and heterozygosity rate > 3SD from the mean. Two control samples had low heterozygosity indicating either divergent ancestry or consanguinity and were excluded from the analysis. The dataset was then filtered to include only autosomal SNPs with a call rate > 98% and a MAF > 2%, and the PLINK[[27](#_ENREF_27)] “—genome” function was used to identify any sample duplicates or related pairs of subjects. Two pairs of samples were identified as > 3^rd^ degree relatives. For each pair, the sample with the lowest SNP call rate was excluded (1 PSP and 1 control). The dataset was further filtered to remove complex genomic regions (chr8:1-12,700,000; chr2:129,900,001-136,800,000; chr17:40,900,001-44,900,000; chr6:32,100,001-33,500,000) and LD pruned using the SNPRelate (v1.4.2) package in R (v3.2.3)[[38](#_ENREF_38)], implementing an LD threshold of 0.15 and a sliding window of 1E-07 bp. The remaining SNPs and subjects were analyzed using EIGENSOFT[[24](#_ENREF_24)] for population outliers. Two samples were identified as population outliers (1 PSP and 1 control) using the default parameters of > 6 SD from the mean on any of the top ten inferred axes following 5 iterations and were removed from further analysis. In total, 2 PSP and 4 control samples were removed from analysis based on genetic data QC, resulting in 82 PSP and 76 control samples analyzed for the Mayo RNASeq cohort.

***Differential Gene Expression (DGE):*** Genes with a median CQN > -1 in both PSP cases and controls were considered “expressed” and CQN expression measures for these 29,880 genes were assessed for differential expression. Multi-variable linear regression implemented using R statistical software was used, including age at death, sex, RIN, brain tissue source, and flowcell as biological and technical covariates. Association p-values were adjusted for multiple tests using false discovery rate (Benjamini-Hochberg)[[31](#_ENREF_31)]. DGE results were linked to gene expression/neuropathological latent trait correlation results using Microsoft Office Access 2007, on a one-to-one basis matching ensemble gene names (DGE) with WG-DASL array gene names (Illumina, San Diego, CA, USA). 14,198 genes measured by RNASeq had overlapping gene names with those assessed for Cohort A microarray data and 12,185 for both Cohorts A and B. Some genes have > 1 probe (WG-DASL) or associated ensemble ID (RNASeq annotation), in these instances both records were linked to the correlation results.

**Module Annotation**

***TreNA*:** DNase Hypersensitivity (DHS) fastq files from ENCODE for all available brain samples was downloaded and aligned using the SNAP method (arXiv:1111.5572v1). Two alignments were performed using seed size 16 and 20 as the sequence data was typically > 50 bp in length. The peak calling algorithm F-seq[[7](#_ENREF_7)] was used to identify regions of open chromatin. Footprinting algorithms for Wellington[[25](#_ENREF_25)] and HINT[[14](#_ENREF_14)] were generated using default parameters. For each individual gene model, footprints within the proximal promoter (+/-5 kb of the transcription start site) are considered as priors in assessing the relationship between the expression of the TF and target gene. Using the R package *trena* (Bioconductor), which utilizes several LASSO regression techniques, Pearson and Spearman correlation and random forest to prioritize a list of putative TF regulators for each gene. Scores from all these approaches were scaled and projected into PCA space and their principle components added together to produce a single composite score (pcaMax). This approach was applied to the transcripts for the larger Cohort_A expression dataset and TFs for the genes in the most significant co-expression networks were identified. The *cis*-eQTL data for the TFs were identified from the Cohort_A eQTL data described above.

***Cell-type enriched genes*:** We identified sets of genes enriched in each of the major cell types in the brain using gene expression measures (RNAseq, FPKM) from purified cell populations, isolated from human brain tissue, obtained from Zhang et al[[37](#_ENREF_37)]. Genes identified to have levels that changed > 4 fold when resected tissue was left for a comparable time to cell isolation protocols (5 hours) were removed from analysis (Supplementary table S2 in Zhang et al[[37](#_ENREF_37)]), as were 512 genes with more than one row of expression data, to avoid ambiguity. We analyzed the expression measures for the remaining 21,390 genes using R Statistical Software to identify sets of genes that are enriched in each of the cell types. For each gene the mean FPKM in the targeted cell type was compared to the mean FPKM for each other cell type and a fold-change calculated; results were filtered to retain genes with a mean FPKM > 5 in the target cell and a fold change > 4 when compared with each other cell type. This approach identified 434 genes enriched in human neurons, 241 genes enriched in human astrocytes, 376 genes enriched in human microglia, 100 genes enriched in human endothelial cells, and 119 genes enriched in human oligodendrocytes; a total of 1,270 unique genes (**suppl. table 26 (Online Resource 1)**). These gene sets were utilized for enrichment analysis (see above).

**Suppl. Results**

**Neuropathology:**

All PSP subjects were assessed by a neuropathologist (DWD) and semi-quantitative measures (counts) were collected for PSP tau neuropathological lesions: CB, NFT, TA and TAUTh across 19 brain regions; an overall score representing all 4 lesions was also generated. Counts were transformed into continuous latent variables as previously described[[2](#_ENREF_2)] and residuals, adjusted for covariates age and sex, were generated prior to analysis (**suppl. figure 1a-b (Online Resource 2), Methods**). To determine the degree of similarity between the 4 neuropathological traits we assessed them for pairwise correlations; we observed positive correlation between each pair (Cohort A: Pearson correlation: 0.24 to 0.60), with NFT and TAUTh having the highest correlation and NFT and TA having the lowest. All four traits were significantly correlated, to various degrees, with the overall trait (Cohort A: Pearson correlation > 0.66); this observation was similar across both microarray cohorts (**suppl. figure 2a-b (Online Resource 2)**).

Since the latent neuropathological traits are generated from semi-quantitative counts from 19 regions (**suppl. figure 2c (Online Resource 2))**, we analyzed a subset of these regions representing all-high neuropathologies (motor cortex, subthalamic nucleus), low TA/high others (medullary tegmentum), low NFT/high others (caudate-putamen) and an all-low neuropathologies region (temporal cortex) for associations with levels of the most significant genes (**suppl. table 1 (Online Resource 1)**). All but one of the genes (*TMEM1*) have more significant latent trait associations than regional neuropathology associations. This is to be expected if the former is informed by multiple regional pathologies that concordantly contribute to the expression associations. Indeed, the directions of association were concordant for most of the regional neuropathologies. Temporal cortex had the least number of nominally significant associations, which would be consistent with the relative lack of neuropathology in this region and provides further support for our rationale of choosing this region for our expression analysis. Medullary tegmentum, which has low TA, had the highest number of nominally significant associations many of which are with NFT trait, which is also consistent with the divergence of TA and NFT neuropathologies with respect to their gene expression associations. In summary, despite the variability in the extent of each neuropathology for the individual brain regions and their associations with brain gene expression levels, there is consistency in the directions for most associations between the different brain regions.

***Expression associations common to all tau lesions*:** Some genes are uniquely associated with just one tau neuropathological trait and may represent genes relevant to tau pathology in the affected cell types. Conversely, genes associated with all four neuropathologic traits may represent key genes involved more generally in tau neuropathology. We identified 91 probes (90 unique genes) that are nominally associated with all four traits (unadjusted p < 0.05) (**figure 1b, suppl. table 7 (Online Resource 1)**), interestingly all of which have a consistent direction of association. Amongst these 90 genes, the most significant for CB is *C20ORF196* (p = 3.96E-06), for NFT is *PRCKB1* (p = 1.68E-06), for TA is *FAM23B* (p = 2.99E-07) and for TAUTh is *CCDC28A* (p = 1.62E-07). *PRCKB1* is a calcium activated serine- and threonine-specific protein kinase with diverse roles; region specific neuronal expression of this gene has been implicated in AD pathogenesis[[12](#_ENREF_12)]. Genetic variation at the *C20ORF196* locus was identified to be associated with reaction time in a GWAS investigating various cognitive phenotypes related to information processing speed[[21](#_ENREF_21)]. Little is currently known about *CCDC28A* or *FAM23B*, however protein-protein interaction networks[[33](#_ENREF_33)] indicate an interaction between *CCDC28A* and *SRPK2* which has been shown to phosphorylate tau[[16](#_ENREF_16)].

***PSP candidate risk gene brain expression levels are associated with PSP neuropathology*:** GWAS have identified common variants associated with altered risk for PSP[[15](#_ENREF_15)], some of which may influence expression of nearby genes[[2](#_ENREF_2), [15](#_ENREF_15)]. To determine if any of the genes near these PSP risk variants at the GWAS loci are associated with tau neuropathology we queried our available results (**suppl. tables 2-6 (Online Resource 1)**) for probes that target genes *in-cis*, defined as located within 100kb of the index PSP risk variants: rs1411478 (Chr1q25.3), rs7571971 (Chr2p11.2), rs1768208 (Chr3p22.1), rs6687758 (Chr1q41), rs2142991 (Chr10q11.21) and rs11568563 (Chr12p12.1). We defined the *cis*-region for risk variants rs8070723 and rs242557 on Chromosome 17q21 as the approximate boundaries of the common 900kb inversion that they tag, due to the extended region of linkage disequilibrium at this locus. For Cohort A we had expression data for 39 *cis*-probes (21 unique genes) and for both WG-DASL cohorts (meta-analysis) we had expression data for 29 *cis*-probes (17 unique genes). Results are provided in **suppl. table 8 (Online Resource 1)**. After considering 39 tests (Bonferonni p-value < 1.28E-03) one probe, ILMN_1680353, *NSF* (Chr17) was significantly associated with Overall, NFT and TAUTh pathology measures, and a further five probes were associated with NFT only. Five of the probes associated with NFT are located in the Chromosome 17q21 inversion, representing *MAPT*, *NSF* and *CRHR1* expression and the sixth represents *IAPP* expression near rs11568563 on chromosome 12. All but one of the significant chromosome 17 probes is positively associated with the neuropathology measures, the exception being ILMN_1800049 (*MAPT*). This probe targets the alternatively spliced exon 6 of the *MAPT* gene, which is absent in the six primary transcripts thought to be expressed in the brain; although differential expression of this exon across different brain regions has been previously reported[[34](#_ENREF_34)]. Given that *MAPT* encodes for tau, a positive correlation between expression of the probe that targets the constitutively expressed 3’ UTR (ILMN_2310814) and tau pathology burden is not unexpected. *CRHR1*, positively associated with NFT, has been linked to stress-induced effects on cognition[[35](#_ENREF_35)]. *NSF,* also positively associated with NFT, plays a key role in intracellular membrane trafficking through interaction with SNARE complexes, important for synaptic transmission and other cellular functions. Recently, it was reported that LRRK2, linked to Parkinson’s disease risk, can phosphorylate NSF resulting in disassembly of the SNARE complex[[5](#_ENREF_5)]. It should be noted that copy number variants at the Chr17q21 locus[[6](#_ENREF_6), [30](#_ENREF_30)] may influence gene expression measures, the precise impact of which requires further evaluation. *IAPP,* negatively correlated with NFT, encodes for the Islet Amyloid Polypeptide, also known as Amylin, which is predominantly expressed in the pancreas and is involved regulation of blood glucose. Amylin aggregates in the brain have been linked to dementia in patients with type II diabetes[[29](#_ENREF_29)]; while it is not immediately clear how decreased brain expression levels of *IAPP* might be linked to increased tau pathology, abnormal glucose metabolism has been implicated in neurodegenerative disease. None of the PSP candidate genes most significantly correlated with pathology measures were also significantly differentially expressed (DE) between PSPs and controls in the RNASeq cohort, however the direction of the DE results is consistent for all but *CRHR1,* and the exon 6 specific probe for *MAPT* (ILMN_1800049) which may not be well represented by the overall RNASeq MAPT expression measure (**suppl. table 8 (Online Resource 1)**).

**Biological characteristics of co-expression modules:** Given the implications of immune system genes in risk for another neurodegenerative disorder, Alzheimer’s disease (AD)[[11](#_ENREF_11)], we also interrogated the “immune system” module, CohortA_M6, which is *positively* associated with TA, for presence of AD risk genes. Of the AD candidate genes near the 20 GWAS loci[[19](#_ENREF_19)], four reside in this “immune system” module, namely, *CD33, INPP5D, MS4A6A* and *PILRA[*[*4*](#_ENREF_4)*]* at the *ZCWPW1* locus. CohortA_M6 also harbors *TYROBP* and *PLCG2*, as the 10^th^ and 18^th^ most well-connected genes, respectively, out of 1,044 transcripts in this module. Both are microglial genes that have been implicated in AD pathophysiology either through brain gene expression network analysis[[36](#_ENREF_36)] or presence of rare coding AD risk variants[[28](#_ENREF_28)]. These findings suggest that altered expression of the “immune system” module may underlie aspects of both AD and PSP pathophysiology.

**Genomic annotations of neuropathology-associated genes and co-expression modules:**

*Hub genes:* We further explored the significant co-expression modules to identify “hub genes” and provide additional annotations (**figure 4**). Hub genes were identified according to two criteria: i. Module membership, which is a measure of the correlation between the individual gene expression and the module eigengene; and ii. Connectivity, which selects for genes with the greatest number of within-module connections, amongst the top 150 connections, defined as the degree of correlation between each pair of genes in the module. Up to 10 top hub genes identified by either of these criteria are provided in **suppl. table 16 (Online Resource 1)**.

Module genes were plotted (**figure 4**) to further visualize the top 150 connections in each of the associated modules and were annotated according to the most significant neuropathological trait correlation and CNS cell type expression signatures (**suppl. table 26 (Online Resource 1)**). For CohortA_M2, 10 hub genes were identified based on the above criteria (**suppl. table 16 (Online Resource 1)**), of which 5 are highly enriched within neurons (*KCNC2, TCEAL6, CDH8, MAP7D2, KRT222*). At least two of these 10 hub genes could have direct relevance to tau neuropathology. *MAP4*, encodes a microtubule associated protein and is a paralog of *MAPT[*[*32*](#_ENREF_32)*]*, which encodes tau. *PTPN3* (PTPH1) is a member of the protein tyrosine phosphatase family, which includes *PTPN1* (PTP1B) that is associated with NFT (**suppl. table 1 (Online Resource 1))** and can influence tau phosphorylation via GSK-3ß[[18](#_ENREF_18)]. Further, some of the hub genes in this module have been implicated in neuropsychiatric diseases and/or neurotransmission. *KCNC2,* which has the highest module membership in CohortA_M2 (**suppl. table 16, suppl. table 20 (Online Resource 1)**), encodes a Shaw family voltage-gated potassium channel important for excitability of membranes, and resides at a locus associated with risk for bipolar disorder (BD)[[1](#_ENREF_1)]. *ERICH3* is known to be highly expressed in the brain and common variants at the *ERICH3* locus have been implicated in a GWAS of plasma serotonin levels[[13](#_ENREF_13)]. We note that there are other genes with high module membership, though not amongst the top 10 hub genes, which are strongly implicated in tau pathophysiology. *CDK5R1* is amongst the most significantly associated genes with NFT (**suppl. table 1 (Online Resource 1)**) and has high connectivity in module CohortA_M2; it encodes the regulatory p35 subunit of cyclin-dependent kinase 5, the truncated form (p25) of which hyperphosphorylates tau and is implicated in neuronal death in neurodegenerative diseases[[10](#_ENREF_10), [23](#_ENREF_23)]. *MAPT* *per se* is also one of the genes in CohortA_M2 that is significantly associated with NFT at q<0.05.

In CohortA_M3, four out of 6 top hub genes include olfactory receptors (**suppl. table 16 (Online Resource 1)**), consistent with the enrichment of “olfactory terms” in this module (**figure 3c,e; figure 4b**). For CohortA_M13 the gene with the highest module membership, *GABPB2*, (**suppl. table 16, suppl. table 18 (Online Resource 1)**), was identified within a differentially methylated region in schizophrenia[[20](#_ENREF_20)], indicating that transcriptional regulation of this gene may play a role in neuropsychiatric disease.

**References:**

1 (2007) Genome-wide association study of 14,000 cases of seven common diseases and 3,000 shared controls. Nature 447: 661-678 Doi 10.1038/nature05911

2 Allen M, Burgess JD, Ballard T, Serie D, Wang X, Younkin CS, Sun Z, Kouri N, Baheti S, Wang Cet al (2016) Gene expression, methylation and neuropathology correlations at progressive supranuclear palsy risk loci. Acta Neuropathol 132: 197-211 Doi 10.1007/s00401-016-1576-7

3 Allen M, Carrasquillo MM, Funk C, Heavner BD, Zou F, Younkin CS, Burgess JD, Chai HS, Crook J, Eddy JAet al (2016) Human whole genome genotype and transcriptome data for Alzheimer's and other neurodegenerative diseases. Sci Data 3: 160089 Doi 10.1038/sdata.2016.89

4 Allen M, Kachadoorian M, Carrasquillo MM, Karhade A, Manly L, Burgess JD, Wang C, Serie D, Wang X, Siuda Jet al (2015) Late-onset Alzheimer disease risk variants mark brain regulatory loci. Neurol Genet 1: e15 Doi 10.1212/NXG.0000000000000012

5 Belluzzi E, Gonnelli A, Cirnaru MD, Marte A, Plotegher N, Russo I, Civiero L, Cogo S, Carrion MP, Franchin Cet al (2016) LRRK2 phosphorylates pre-synaptic N-ethylmaleimide sensitive fusion (NSF) protein enhancing its ATPase activity and SNARE complex disassembling rate. Mol Neurodegener 11: 1 Doi 10.1186/s13024-015-0066-z

6 Boettger LM, Handsaker RE, Zody MC, McCarroll SA (2012) Structural haplotypes and recent evolution of the human 17q21.31 region. Nat Genet 44: 881-885 Doi 10.1038/ng.2334

7 Boyle AP, Guinney J, Crawford GE, Furey TS (2008) F-Seq: a feature density estimator for high-throughput sequence tags. Bioinformatics 24: 2537-2538 Doi 10.1093/bioinformatics/btn480

8 Braak H, Braak E (1991) Neuropathological stageing of Alzheimer-related changes. Acta Neuropathol 82: 239-259

9 Carrasquillo MM, Zou F, Pankratz VS, Wilcox SL, Ma L, Walker LP, Younkin SG, Younkin CS, Younkin LH, Bisceglio GDet al (2009) Genetic variation in PCDH11X is associated with susceptibility to late-onset Alzheimer's disease. Nat Genet 41: 192-198 Doi 10.1038/ng.305

10 Cruz JC, Tseng HC, Goldman JA, Shih H, Tsai LH (2003) Aberrant Cdk5 activation by p25 triggers pathological events leading to neurodegeneration and neurofibrillary tangles. Neuron 40: 471-483

11 Efthymiou AG, Goate AM (2017) Late onset Alzheimer's disease genetics implicates microglial pathways in disease risk. Mol Neurodegener 12: 43 Doi 10.1186/s13024-017-0184-x

12 Gerschutz A, Heinsen H, Grunblatt E, Wagner AK, Bartl J, Meissner C, Fallgatter AJ, Al-Sarraj S, Troakes C, Ferrer Iet al (2014) Neuron-specific alterations in signal transduction pathways associated with Alzheimer's disease. J Alzheimers Dis 40: 135-142 Doi 10.3233/JAD-131280

13 Gupta M, Neavin D, Liu D, Biernacka J, Hall-Flavin D, Bobo WV, Frye MA, Skime M, Jenkins GD, Batzler Aet al (2016) TSPAN5, ERICH3 and selective serotonin reuptake inhibitors in major depressive disorder: pharmacometabolomics-informed pharmacogenomics. Mol Psychiatry 21: 1717-1725 Doi 10.1038/mp.2016.6

14 Gusmao EG, Allhoff M, Zenke M, Costa IG (2016) Analysis of computational footprinting methods for DNase sequencing experiments. Nat Methods 13: 303-309 Doi 10.1038/nmeth.3772

15 Hoglinger GU, Melhem NM, Dickson DW, Sleiman PM, Wang LS, Klei L, Rademakers R, de Silva R, Litvan I, Riley DEet al (2011) Identification of common variants influencing risk of the tauopathy progressive supranuclear palsy. Nat Genet 43: 699-705 Doi 10.1038/ng.859

16 Hong Y, Chan CB, Kwon IS, Li X, Song M, Lee HP, Liu X, Sompol P, Jin P, Lee HGet al (2012) SRPK2 phosphorylates tau and mediates the cognitive defects in Alzheimer's disease. J Neurosci 32: 17262-17272 Doi 10.1523/JNEUROSCI.3300-12.2012

17 Kalari KR, Nair AA, Bhavsar JD, O'Brien DR, Davila JI, Bockol MA, Nie J, Tang X, Baheti S, Doughty JBet al (2014) MAP-RSeq: Mayo Analysis Pipeline for RNA sequencing. BMC Bioinformatics 15: 224 Doi 10.1186/1471-2105-15-224

18 Kanno T, Tsuchiya A, Tanaka A, Nishizaki T (2016) Combination of PKCepsilon Activation and PTP1B Inhibition Effectively Suppresses Abeta-Induced GSK-3beta Activation and Tau Phosphorylation. Mol Neurobiol 53: 4787-4797 Doi 10.1007/s12035-015-9405-x

19 Lambert JC, Ibrahim-Verbaas CA, Harold D, Naj AC, Sims R, Bellenguez C, DeStafano AL, Bis JC, Beecham GW, Grenier-Boley Bet al (2013) Meta-analysis of 74,046 individuals identifies 11 new susceptibility loci for Alzheimer's disease. Nat Genet 45: 1452-1458 Doi 10.1038/ng.2802

20 Lee SA, Huang KC (2016) Epigenetic profiling of human brain differential DNA methylation networks in schizophrenia. BMC Med Genomics 9: 68 Doi 10.1186/s12920-016-0229-y

21 Luciano M, Hansell NK, Lahti J, Davies G, Medland SE, Raikkonen K, Tenesa A, Widen E, McGhee KA, Palotie Aet al (2011) Whole genome association scan for genetic polymorphisms influencing information processing speed. Biol Psychol 86: 193-202 Doi 10.1016/j.biopsycho.2010.11.008

22 Mirra SS, Gearing M, McKeel DW, Jr., Crain BJ, Hughes JP, van Belle G, Heyman A (1994) Interlaboratory comparison of neuropathology assessments in Alzheimer's disease: a study of the Consortium to Establish a Registry for Alzheimer's Disease (CERAD). J Neuropathol Exp Neurol 53: 303-315

23 Patrick GN, Zukerberg L, Nikolic M, de la Monte S, Dikkes P, Tsai LH (1999) Conversion of p35 to p25 deregulates Cdk5 activity and promotes neurodegeneration. Nature 402: 615-622 Doi 10.1038/45159

24 Patterson N, Price AL, Reich D (2006) Population structure and eigenanalysis. PLoS Genet 2: e190 Doi 10.1371/journal.pgen.0020190

25 Piper J, Elze MC, Cauchy P, Cockerill PN, Bonifer C, Ott S (2013) Wellington: a novel method for the accurate identification of digital genomic footprints from DNase-seq data. Nucleic Acids Res 41: e201 Doi 10.1093/nar/gkt850

26 Price AL, Patterson NJ, Plenge RM, Weinblatt ME, Shadick NA, Reich D (2006) Principal components analysis corrects for stratification in genome-wide association studies. Nat Genet 38: 904-909 Doi 10.1038/ng1847

27 Purcell S, Neale B, Todd-Brown K, Thomas L, Ferreira MA, Bender D, Maller J, Sklar P, de Bakker PI, Daly MJet al (2007) PLINK: a tool set for whole-genome association and population-based linkage analyses. Am J Hum Genet 81: 559-575

28 Sims R, van der Lee SJ, Naj AC, Bellenguez C, Badarinarayan N, Jakobsdottir J, Kunkle BW, Boland A, Raybould R, Bis JCet al (2017) Rare coding variants in PLCG2, ABI3, and TREM2 implicate microglial-mediated innate immunity in Alzheimer's disease. Nat Genet 49: 1373-1384 Doi 10.1038/ng.3916

29 Srodulski S, Sharma S, Bachstetter AB, Brelsfoard JM, Pascual C, Xie XS, Saatman KE, Van Eldik LJ, Despa F (2014) Neuroinflammation and neurologic deficits in diabetes linked to brain accumulation of amylin. Mol Neurodegener 9: 30 Doi 10.1186/1750-1326-9-30

30 Steinberg KM, Antonacci F, Sudmant PH, Kidd JM, Campbell CD, Vives L, Malig M, Scheinfeldt L, Beggs W, Ibrahim Met al (2012) Structural diversity and African origin of the 17q21.31 inversion polymorphism. Nat Genet 44: 872-880 Doi 10.1038/ng.2335

31 Storey JD, Tibshirani R (2003) Statistical significance for genome-wide experiments. . Proceeding of the National Academy of Sciences 100 9440-9445

32 Sundermann F, Fernandez MP, Morgan RO (2016) An evolutionary roadmap to the microtubule-associated protein MAP Tau. BMC Genomics 17: 264 Doi 10.1186/s12864-016-2590-9

33 Szklarczyk D, Franceschini A, Wyder S, Forslund K, Heller D, Huerta-Cepas J, Simonovic M, Roth A, Santos A, Tsafou KPet al (2015) STRING v10: protein-protein interaction networks, integrated over the tree of life. Nucleic Acids Res 43: D447-452 Doi 10.1093/nar/gku1003

34 Trabzuni D, Wray S, Vandrovcova J, Ramasamy A, Walker R, Smith C, Luk C, Gibbs JR, Dillman A, Hernandez DGet al (2012) MAPT expression and splicing is differentially regulated by brain region: relation to genotype and implication for tauopathies. Hum Mol Genet 21: 4094-4103 Doi 10.1093/hmg/dds238

35 Wang XD, Chen Y, Wolf M, Wagner KV, Liebl C, Scharf SH, Harbich D, Mayer B, Wurst W, Holsboer Fet al (2011) Forebrain CRHR1 deficiency attenuates chronic stress-induced cognitive deficits and dendritic remodeling. Neurobiol Dis 42: 300-310 Doi 10.1016/j.nbd.2011.01.020

36 Zhang B, Gaiteri C, Bodea LG, Wang Z, McElwee J, Podtelezhnikov AA, Zhang C, Xie T, Tran L, Dobrin Ret al (2013) Integrated systems approach identifies genetic nodes and networks in late-onset Alzheimer's disease. Cell 153: 707-720 Doi 10.1016/j.cell.2013.03.030

37 Zhang Y, Sloan SA, Clarke LE, Caneda C, Plaza CA, Blumenthal PD, Vogel H, Steinberg GK, Edwards MS, Li Get al (2016) Purification and Characterization of Progenitor and Mature Human Astrocytes Reveals Transcriptional and Functional Differences with Mouse. Neuron 89: 37-53 Doi 10.1016/j.neuron.2015.11.013

38 Zheng X, Levine D, Shen J, Gogarten SM, Laurie C, Weir BS (2012) A high-performance computing toolset for relatedness and principal component analysis of SNP data. Bioinformatics 28: 3326-3328 Doi 10.1093/bioinformatics/bts606

39 Zou F, Chai HS, Younkin CS, Allen M, Crook J, Pankratz VS, Carrasquillo MM, Rowley CN, Nair AA, Middha Set al (2012) Brain expression genome-wide association study (eGWAS) identifies human disease-associated variants. PLoS Genet 8: e1002707 Doi 10.1371/journal.pgen.1002707
